# Mathematical modelling of telomere length dynamics

**DOI:** 10.1007/s00285-019-01448-y

**Published:** 2019-11-14

**Authors:** Jonathan A. D. Wattis, Qi Qi, Helen M Byrne

**Affiliations:** 1 School of Mathematical Sciences, University of Nottingham, Nottingham, NG7 2RD UK; 2 Mathematics Institute, University of Oxford, Andrew Wiles Building, Radcliffe Observatory Quarter, Woodstock Road, Oxford, OX2 6GG UK

**Keywords:** Telomere dynamics, End-replication problem, Aging, Mathematical, 35Q92, 35B40, 92C37, 35Kxx, 35Q62

## Abstract

Telomeres are repetitive DNA sequences located at the ends of chromosomes. During cell division, an incomplete copy of each chromosome’s DNA is made, causing telomeres to shorten on successive generations. When a threshold length is reached replication ceases and the cell becomes ‘senescent’. In this paper, we consider populations of telomeres and, from discrete models, we derive partial differential equations which describe how the distribution of telomere lengths evolves over many generations. We initially consider a population of cells each containing just a single telomere. We use continuum models to compare the effects of various mechanisms of telomere shortening and rates of cell division during normal ageing. For example, the rate (or probability) of cell replication may be fixed or it may decrease as the telomeres shorten. Furthermore, the length of telomere lost on each replication may be constant, or may decrease as the telomeres shorten. Where possible, explicit solutions for the evolution of the distribution of telomere lengths are presented. In other cases, expressions for the mean of the distribution are derived. We extend the models to describe cell populations in which each cell contains a distinct subpopulation of chromosomes. As for the simpler models, constant telomere shortening leads to a linear reduction in telomere length over time, whereas length-dependent shortening results in initially rapid telomere length reduction, slowing at later times. Our analysis also reveals that constant telomere loss leads to a Gaussian (normal) distribution of telomere lengths, whereas length-dependent loss leads to a log-normal distribution. We show that stochastic models, which include a replication probability, also lead to telomere length distributions which are skewed.

## Introduction

Repetitive DNA sequences at the end of chromosomes—known as telomeres—are shortened when cells divide, leading to one aspect of cellular aging. In this paper we derive and analyse mathematical models which describe the evolution of the distribution of telomere lengths over many generations of the cell-cycle.

In the 1960s Hayflick and Moorehead (1961) performed a series of experiments which overturned the prevailing view that normal cells were immortal. In experiments involving normal human fibroblasts cells, they found that after a finite number of divisions cell numbers reached a finite size, which is now called *the Hayflick limit*. Once this limit is reached, the cells become senescent: that is, they stop replicating but remain functional (Cristofalo et al. 2004). In later work, Hayflick and Moorehead observed that, the number of senescent cells in the mouse lens epithelium increases with age. Processes that contribute to senescence include oxidative stress, as shown by Muller et al. (2007) and Wei and Lee (2002); mitochondrial dysfunction (Passos and von Zglinicki 2005); somatic mutation (Kirkwood and Proctor 2003); and, of particular relevance here, telomere shortening, which was demonstrated by Allsopp and Harley (1995).

**Fig. 1:**
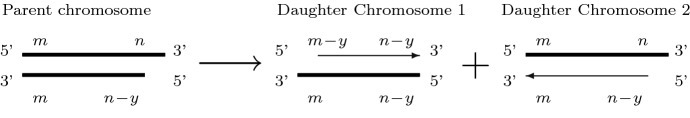
Illustration of the chromosome replication process: on the left, the two strands of parent DNA are shown, with telomeres of lengths *m*, *m*, *n* and $n-y$; the two offspring chromosomes are shown to the right of the arrow. In each case the strand inherited from the parent is shown with a thick line, and the thin lines show the newly synthesised strand, with the arrow indicating the direction of synthesis. Daughter chromosome 2 is seen to be identical to the parent; having inherited the longer strand from the parent, and synthesised the shorter strand. However, daughter chromosome 1 inherits the shorter strand of parent DNA, and synthesises an even shorter complementary strand, resulting in telomeres of lengths *m*, $m-y$, $n-y$ and $n-y$

The main roles of telomeres are to protect the chromosomes against the loss of genetic material and to prevent fragments of chromosomes from rejoining (Cooper and Hausman 2009). Kirkwood (2011) has given compelling evidence that telomeres play an important role in ageing, with telomere length being a key factor in determining a cell’s replicative potential. When a cell divides, its chromosomes are duplicated *via* a process called DNA replication. During replication, one of the daughter chromosomes is shortened at the 5’ end due to the unidirectional synthesis of the new DNA chain (Olovnikov 1973). Figure 1 illustrates the effect of this process, where *m*, *n* describe the lengths of telomeres at each end of the chromosome. This process continues until the telomere length falls below a critical level and then the cell becomes senescent. We use the terms ‘telomere loss’ and ‘telomere-shortening’ interchangeably, to refer to the reduced length of telomeres in daughter cells when compared to the parent cell.

In healthy human cells, telomeres are typically 3 to 15 kilobasepairs (kbp) in length. They shorten at rates of 50–200 basepairs per replication, and undergo 30–60 population doublings before becoming senescent (Harley et al. 1990). When the chromosomes become too short, that is, their length falls below a threshold value, telomeres lose their protective function, triggering DNA damage which can lead to end-to-end fusions, chromosome breakage, rejoins and senescence. Evidence that telomere shortening is directly related to cell senescence can be traced back to experiments by Allsopp and Harley (1995) in which the telomeres of senescent human fibroblasts were found to be shorter than those of replicating cells and the proportion of replicating cells was proportional to the mean telomere length. Under certain conditions, the amount of telomere loss per replication is large, and cells become senescent more rapidly. For example, Wyllie et al. (2000) have shown that excessive telomere shortening associated with Werner’s syndrome causes patients to experience accelerated aging. In other cases, where the enzyme telomerase is active, as in cancer cells, telomere length can be maintained or even extended, enabling the cells to become immortal (Greider and Blackburn 1985). von Zglinicki et al. (2000) suggest that there are multiple mechanisms which contribute to telomere shortening, including oxidative stress. By dosing cells with hydrogen peroxide, they showed that oxidative stress accelerated telomere loss and led to shorter replicative lifespans. The effect of perceived life stress and stress due to being a long-term care-giver on telomere length has been analysed by Epel et al. (2004), who showed that increased stress correlates with reduced telomere length. We conclude that normal ageing can be characterised by telomere shortening and that certain environmental and genetic factors may accelerate telomere shortening.

### Simulations and mathematical models of telomere loss

Werner et al. (2015) have fitted tissue-level models to data from subjects from birth to age 85 years. In their models of cellular differentiation and telomere shortening, cells are assumed to lose a fixed length of telomere per replication; and stem cells, divide either asymmetrically (giving rise to another stem cell and a cell with shorter telomeres) or symmetrically (where both daughter cells have shorter telomeres. Fitting their model to experimental data, they obtain good agreement with the observed decreasing rate of telomere loss over time.

Telomeres are thought to adopt the G-quadruplex structure at the 3’-end, which affects the end replication of DNA. This alters the susceptibility to the alternative lengthening of telomere (ALT) mechanism (Tan et al. 2015). Recent theoretical work of Bodova et al. (2012), Kollar et al. (2014) and Hirt et al. (2014) have focused on understanding the detailed dynamics associated with these structures and processes.

Various models of telomere shortening in a population of independently replicating chromosomes have been proposed previously: Levy et al. (1992) developed a deterministic model for telomere shortening of individual chromosomes. Their model shows good agreement with experimental data, predicting that the average telomere length decreases linearly over time. In separate work, Arino et al. (1995) assumed constant telomere loss per replication and viewed cell proliferation as a branching process. A convincing fit of their model to independent experimental data is provided. Olofsson and Kimmel (1999) extended Arino’s model to account for cell death, assuming a fixed probability of cell death for senescent cells.

While many mechanisms are known to cause telomere shortening, the *rate* of shortening remains unclear. Many mathematical models assume a constant rate of telomere loss, for example, Levy et al. (1992). In their model, however, Buijs et al. (2004) assumed that telomere loss depends linearly on telomere length: a constant loss is attributed to the end-replication problem and an additional term is attributed to a shortening factor which they estimate from experimental data. Portugal et al. (2008) took a different approach, developing a *stochastic* model in which telomere shortening occurs at a constant rate but the *probability* of cell division depends linearly on the telomere length. The resulting stochastic model is well-described at the population level by a Gompertzian growth law.

### Outline

This paper is structured as follows: in Sect. 1.1 we summarise previous theoretical work on telomere shortening. In Sect. 1.3 we outline our modelling approach. This is based on deriving equations for the distribution of telomere lengths in a population of chromosomes or cells which are repeatedly undergoing replication, causing their telomeres to shorten over time. In Sects. 2, 3, 4 and 5 we investigate four such models: in the first two, we consider populations of chromosomes that replicate independently of each other. In Sects. 4 and 5, the earlier models are scaled up to the cell level: we consider populations of cells, where each cell comprises $N=46$ chromosomes. This model extension enables us to investigate how the lengths of subpopulations of telomeres evolve in cell populations. In all the models we develop, cells are assumed to exist in either a replicative or a senescent state. Once senescent, a cell remains senescent forever; we do not model cell death. Finally, in Sect. 6 we summarise the results and draw conclusions.

In each of Sects. 2, 3, 4 and 5, we start with a discrete model which describes how the numbers of cells with a particular telomere length changes from one generation, *g*, to the next. Since we are interested in the evolution over many generations, where all numbers will be large and, in general, the amount of telomere lost per replication is significantly smaller than the telomere length, it is appropriate to replace all these quantities with continuous variables. By considering the evolution of slowly-varying solutions of the discrete equation, we derive continuum models, namely partial differential equations (pdes) which have the same dynamics. The advantage of this approach is that, in general, the resulting pdes are more amenable to theoretical analysis than discrete systems. We use asymptotic techniques to construct and analyse solutions of the resulting pdes.

Our approach unifies and extends existing models: in particular, our approach includes, deterministic approaches where telomere loss is constant (Levy et al. 1992), dependent on telomere length (Buijs et al. 2004), as well as stochastic models, in which there is a probability of cell-replication which depends on telomere length, as considered by Portugal et al. (2008). Our aim is to explain the way in which telomere shortening occurs leads to senescence, for example, to predict how the fraction of a population which is senescent changes over time, and to determine whether it is possible to deduce from such data the mechanisms that regulate telomere shortening. We present results which show the approximate solutions of the pdes are in good agreement with numerical simulations from the earlier paper by Qi et al. (2014).

### Preliminary concepts of model development

Our main aim in this paper is to analyse mathematical models which describe the distribution of telomere lengths and their evolution over many generations. Our models are based on the earlier work of Levy et al. (1992), Buijs et al. (2004), and Portugal et al. (2008).

Initially we view the generation number as a discrete variable which we denote by *g*. We denote the telomere length of a chromosome by a single variable, *n* measured as a number of base pairs. Typically, telomeres are initially long ($\sim$ 6000 bps) and lose only a small number of base pairs during each replication event ($\sim$ 50 bps). Denoting by $K_n^{(g)}$ a chromosome at generation *g* with telomere length *n*, and the number of basepairs lost upon replication by *y*(*n*), the process of replication can be described by

1.1 $$\begin{aligned} K_{n}^{(g)}\rightarrow K_{n}^{(g+1)} +K_{n-y(n)}^{(g+1)}\,. \end{aligned}$$

In Eq. (1.1), we assume that replication of a chromosome with telomere length *n* at generation *g*, produces two ‘daughter’ chromosomes at generation $g+1$, one with telomere length *n* and one with length $n-y(n)$. If the length of the daughter chromosome $n-y(n)$ falls below a prescribed, critical value, then replication will not happen. The value of $n_c$ will be specified later.

In our analysis, we follow Buijs et al. (2004) and assume that

1.2 $$\begin{aligned} y(n) = y_{0} + n y_1 , \end{aligned}$$

with $y_0,y_1$ nonnegative constants, so that the number of base pairs lost is positive and may depend on telomere length. The case of Levy et al. (1992), where telomere loss is constant, is recovered by setting $y_1=0$. In general we take $y_1>0$ so that longer telomeres shorten at a faster rate than shorter ones; however, the analysis presented below is valid for the case where longer telomeres have a lower rate of loss ($y_1<0$) provided no lengthening of telomeres occurs ($y(n) \ge 0$, that is, $n<-y_0/y_1=y_0/|y_1|$).

We term the deterministic model given by (1.1) as Case A and analyse it in Sect. 2 below. In Sect. 3 we generalise it to a stochastic model, termed Case B, by assuming that replication is a stochastic event which occurs with probability $P_{\mathrm{div}}(n)$ so that the telomere replication rate depends on its length, *n*; that is, (1.1) occurs with probability $P_{\mathrm{div}}(n)$, and otherwise, we simply have $K_n^{(g)} \rightarrow K_n^{(g+1)}$. We define the division probability as

1.3 $$\begin{aligned} P_{\mathrm{div}}(n) = a n + b , \end{aligned}$$

where *a*, *b* are constants, chosen such that $0 \le P_{\mathrm{div}} \le 1$ for telomere lengths, *n*, in the range of interest. Hence for Case B, the replication rule (1.1) is replaced by

1.4 $$\begin{aligned} K_{n}^{(g)}\rightarrow \left\{ \begin{array}{ll} K_{n}^{(g+1)} +K_{n-y(n)}^{(g+1)}\, &{} \qquad \hbox {with probability } P_{\mathrm{div}}(n) , \\ K_{n}^{(g+1)} &{} \qquad \text{ otherwise. } \end{array}\right. \end{aligned}$$

The deterministic Case A can be viewed as a special case of Case B, for which $P_{\mathrm{div}}(n)=1$.

**Table 1:** Summary of the functional forms used to model cell division ($P_{\mathrm{div}}(n)$) and telomere shortening (*y*(*n*)) where $n \ge 0$ represents the telomere length of a particular chromosome and $y_{0}$, $y_1$, *a*, *b* and $\alpha$ are non-negative constants

Model	Replication rule	Shortening rule	References
Case A	$P_{\mathrm{div}}=1$	$y(n)=y_{0}+y_1n$	Buijs et al. (2004)
			Levy et al. (1992) ($y_1 = 0$)
Case B	$P_{\mathrm{div}}(n) = a n + b$	$y(n)=y_{0}+y_1n$	Qi et al. (2014)
			Portugal et al. (2008) ($y_1 = 0$)

In the following sections, we describe and analyse the models summarised in Table 1. In Sect. 2 we start with Case A, which describes a population of chromosomes replicating independently.

## Case A: deterministic models of telomere shortening in individual chromosomes

### Formulation of discrete model

In this section we use the replication rule (1.1) to derive a discrete dynamical system which predicts how the distribution of telomere lengths evolves over the generations. More precisely, we propose a discrete model which relates the number of telomeres of length *n* ($n\ge 0$) at generation $(g + 1)$ to the number of telomeres lengths *n* and $\widetilde{n}$ (where $\widetilde{n} -y(\widetilde{n})=n$ at generation *g*. In general, the amount of telomere lost depends only on the current telomere length. We generate a continuum limit from the discrete system by assuming that the distribution varies slowly with respect to both generation number *g* and telomere length *n*. Finally, we construct solutions of the continuum model and discuss the properties of the solutions.

We start by assuming that replication occurs with probability $P_{\mathrm{div}}=1$ and that the number of basepairs lost during replication depends on telomere length *via* (1.2). The replication rule (1.1) can then be written as

2.1 $$\begin{aligned} K_{n}^{(g)}\rightarrow K_{n}^{(g+1)} +K_{n-(y_{0}+y_{1}n)}^{(g+1)}\, , \qquad \left( n > n_c := \frac{y_0}{1-y_1}\right) . \end{aligned}$$

Thus chromosomes of length *n* at generation $(g+1)$ arise from parent telomeres with the same length, *n*, or a slightly longer telomere, of length $(n+y_0)/(1-y_1) > n$. We remark that if $n<n_c$, then (2.1) would produce a daughter chromosome with a negative telomere length. Since negative telomere lengths are not physically realistic, we do not allow such events to occur. In particular, if $0 \le n \le n_c$ then we assume that replication does not occur, and in place of (2.1) we have $K_n^{(g)} \rightarrow K_n^{(g+1)}$ so that the existing chromosome remains in the population, but does not undergo replication. Such chromosomes are termed ‘senescent’. Identical results could be obtained by allowing (2.1) to occur for all *n*, but only consider $n\ge 0$, when analysing the solution for $K_n^{(g)}$. The parameters and their ranges are summarised in Table 2.

We now introduce $\widetilde{K}_n^{(g)}$ to denote the *number* of chromosomes with telomere length *n* at generation *g*, that is, $\widetilde{K}_n^{(g)}$ represents the concentration of telomere $K_n^{(g)}$. Hence we model the process (2.1) *via* the equation

2.2 $$\begin{aligned} \widetilde{K}_{n}^{(g+1)} = \widetilde{K}_{n}^{(g)} +\widetilde{K}_{\frac{n+y_{0}}{1-y_{1}}}^{(g)}\,,\qquad (n\ge 0). \end{aligned}$$

Typically, we assume that initially ($g=0$), there is just one chromosome with a telomere length of *Q* basepairs, so that

2.3 $$\begin{aligned} \widetilde{K}_n^{(0)}=\delta _{n,Q}, \end{aligned}$$

where $\delta _{n,Q}$ is the Kronecker delta function ($\delta _{n,Q}=1$ if $n=Q$, and $\delta _{n,Q}=0$ otherwise). The early generations ($g=\mathcal {O}(1)$) constitute a transient timescale during which the distribution evolves from the initial condition (2.3) to a smooth distribution. The effect of replication rule (2.1) on the distribution of telomere lengths is localised, causing the distribution to spread out (1-sided) and the mean telomere length to fall. In the next section, from (2.2) we develop a second order pde approximation, which will need two boundary conditions to be applied at small and large *n*. Thus it is helpful to note that the initial conditions (2.3) and evolution Eq. (2.2) discussed above together imply that $K_n^{(g)}=0$ for $n<0$ and $K_n^{(g)}=0$ for $n>Q$.

**Table 2:** Summary of definitions of parameters and variables together with approximate ranges

Parameter or variable	Range of values	Relative magnitude	Brief description
*Q*	(3000, 15000)	$\gg 1$	Maximum telomere length
*L*	$(0,\frac{1}{30})$	$\ll 1$	Typical fraction of telomere lost per generation
$y_0$	(0, 200)	*LQ*	Length-independent rate of loss of telomere
$y_1$	$(0,\frac{1}{30})$	*L*	Coefficient for length-dependent telomere loss
*a*	(0, 1 / *Q*)	1 / *Q*	Coefficient for length-dependence in $P_{\mathrm{div}}$
*b*	$(-1,1)$	$\mathcal {O}(1)$	Length-independent component of $P_{\mathrm{div}}$
*n*	(0, *Q*)	$\mathcal {O}(Q)$	Telomere length
*g*	(0, 1 / *L*)	$\mathcal {O}(1/L)$	Generation number
*x*	$(\log y_0,\log (y_0 + y_1Q))$	$\mathcal {O}(1)$	Rescaled measure of telomere length
$\tau$	(0, 2)	$\mathcal {O}(1)$	Rescaled measure of time

### Derivation of continuum model

In normal human cells, telomeres range in length from 3 to 15k basepairs and the average length of telomere lost during chromosome replication is 50–200 basepairs (Harley et al. 1990), which is much less than the initial telomere length. Thus we define *Q* to be the maximum telomere length, and introduce the small parameter $L\ll 1$ to represent the typical fraction of telomere lost per generation (per chromosome).

Since the relative length of telomere lost per generation is $\mathcal{O}(10^{-2})$, we treat telomere length (*n*) as a continuous variable. For the special case $y_1=0$, telomere loss $y(n)=y_0$ occurs at a constant rate (2.1), and we define

2.4 $$\begin{aligned} x=n/Q , \quad \text{ and } \;\;\; L=y_0/Q \ll 1 , \end{aligned}$$

so that $x = \mathcal {O}(1)$ and the governing evolution Eq. (2.2) can be rewritten as

2.5 $$\begin{aligned} \widetilde{K}_n^{(g+1)} = \widetilde{K}_n^{(g)} + \widetilde{K}_{n+y_0}^{(g)} . \end{aligned}$$

We introduce the cumulative distribution function $G_n^{(g)}$ defined by $\widetilde{K}_n^{(g)} = G_n^{(g)} - G_{n-1}^{(g)}$, which is equivalent to $G_n^{(g)}=\sum _{q=0}^n\widetilde{K}_q^{(g)}$. From (2.5), we have

2.6 $$\begin{aligned} G_n^{(g+1)} = G_n^{(g)} + G_{n+y_0}^{(g)} , \end{aligned}$$

with initial data (at generation $g=0$) of $G_{n}^{(0)} = H(n-Q)$, where $H(\cdot )$ is the Heaviside function ($H(x)=1$ if $x>0$ and $H(x)=0$ if $x<0$).

To account for the expected exponential growth in the population, we write

2.7 $$\begin{aligned} G_n^{(g)} = 2^g F(x,\tau ), \end{aligned}$$

where $F(x,\tau )$ is a cumulative distribution function satisfying the initial and boundary conditions $F(x,0)=H(x-1)$, $F(0,\tau )=0$ and $F(x,\tau )\rightarrow 1$ as $x\rightarrow +\infty$. The boundary conditions correspond to zero-flux conditions which ensure that telomeres of length greater than *Q* and less than zero cannot be formed. Using (2.7) together with (2.4), Eq. (2.6) can be rewritten as

2.8 $$\begin{aligned} 2 F(x,\tau +L) = F(x,\tau ) + F(x+L,\tau ) . \end{aligned}$$

Since the term in (2.8) at $\tau +L$ has a coefficient of two, and the terms at $\tau$ have a combined coefficient of two, it is natural to expand about $\tau +\frac{1}{2}L$. And since the term at $x+L$ only has a weighting of one, whereas the terms at *x* together have coefficients summing to three, the ‘centre of mass’ of the template (2.8) occurs at $x+\frac{1}{4}L$. Thus the natural point about which to perform a Taylor series expansion of (2.8) is $(x+\frac{1}{4} L, \tau +\frac{1}{2}L)$, and performing this expansion yields the pde

2.9 $$\begin{aligned} F_\tau = \frac{1}{2}F_x + \frac{1}{8} L F_{xx} . \end{aligned}$$

By expanding about $(x + \frac{1}{4} L, \tau + \frac{1}{2}L)$, the coefficients of the terms involving $F_{\tau \tau }$ and $F_{x\tau }$ vanish and we obtain the pde. We aim to solve (2.9) subject to the boundary conditions (2.18).

An alternative derivation of (2.9) is available *via* asymptotic analysis: we note that the leading order approximation of (2.8) for $L\ll 1$ is $F_\tau = \frac{1}{2}F_x$, which has a travelling wave solution of the form $F(x,\tau )=\widehat{F}(z,T)$ where $z=x+\frac{1}{2}\tau$ and *T* is a new, longer, timescale. To include the next order correction terms, we introduce

2.10 $$\begin{aligned} z=x+\frac{1}{2}\tau , \quad T=L\tau , \quad \widehat{F}(z,T)=F(x,\tau ), \end{aligned}$$

so that (2.8) can be rewritten as

2.11 $$\begin{aligned} {2} \widehat{F}\left( z+\frac{1}{2}L ,T+L^2\right) = \widehat{F}(z,T) + \widehat{F}(z+L,T) . \end{aligned}$$

A Taylor expansion of this equation about (*z*, *T*) gives the pde$\widehat{F}_T = \frac{1}{8} \widehat{F}_{zz}$, which, when converted back from (*z*, *T*) to $(x,\tau )$, leads to (2.9).

Another method for deriving the continuum limit of (2.8) relies on considering the behaviour of solutions which are slowly-varying in both *x* and $\tau$. Since (2.8) and equations of the form $F_\tau = c F_x + \phi L F_{xx}$ are both linear, each possess solutions of the form $F(x,\tau ) = \mathrm{e}^{-\gamma \tau - \beta x}$, where the relationships between $\beta$ and $\gamma$ are respectively

2.12 $$\begin{aligned} 2\mathrm{e}^{-L\gamma } = 1 + \mathrm{e}^{-L\beta } , \qquad \text{ and } \quad \gamma = c \beta - \phi L \beta ^2 . \end{aligned}$$

Rearranging the former, and expanding for $L\ll 1$, we obtain

2.13 $$\begin{aligned} \gamma = \frac{-1}{L} \log \left( \frac{ 1+\mathrm{e}^{-\beta L} }{2} \right) \sim \frac{1}{2}\beta - \frac{1}{8} \beta ^2 L . \end{aligned}$$

Equating this with the second expression in (2.12), we obtain $c=\frac{1}{2}$, $\phi =\frac{1}{8}$ and thus $F_\tau = \frac{1}{2}F_x +\frac{1}{8}LF_{xx}$ as the appropriate approximation of (2.8).

### The general continuum model

To describe the evolution of the distribution in the *general* case $y_1\ne 0$, we introduce a new, continuous variable for telomere length, *x*(*n*), and a new timescale, $\tau$, defined, respectively, by

2.14 $$\begin{aligned} x=x(n) = \log (y_0+y_1 n), \qquad \tau =Lg , \qquad L:=-\log (1-y_1) \sim y_1 . \end{aligned}$$

The motivation for the introduction of *x*(*n*) as a measure of telomere length is that under (2.1) a telomere of length *x*(*n*) gives rise to offspring of lengths *x*(*n*) and $\widetilde{x}(n)$, where

2.15 $$\begin{aligned} \widetilde{x}(n)= & {} x(n - y_0-y_1n) = \log ( y_0 + y_1 n - y_0 y_1 - y_1^2 n ) \nonumber \\= & {} \log ((1 - y_1)(y_0 + y_1n)) = \log (y_0 + y_1n) + \log (1 - y_1) = x(n) - L.\nonumber \\ \end{aligned}$$

Thus we obtain a model in which the telomere loss is no longer dependent on the telomere length, *x*. Defining $\widehat{K}^{(g)}(x) =\widetilde{K}^{(g)}_n$, in place of (2.2), we have

2.16 $$\begin{aligned} \widehat{K}^{(g+1)}(x) = \widehat{K}^{(g)}(x) + \widehat{K}^{(g)}(x+L) . \end{aligned}$$

If we introduce the cumulative distribution $G^{(g)}(x)$ defined by $\widehat{K}^{(g)}(x)=\mathrm{d}G^{(g)}/\mathrm{d}x$, which is equivalent to $G^{(g)}(x) = \int _{q=0}^{x} \widehat{K}^{(g)}(q)\,\mathrm{d}q$, then we obtain

2.17 $$\begin{aligned} G^{(g+1)}(x) = G^{(g)}(x) + (1-y_1) G^{(g)} \left( \frac{x+y_0}{1-y_1} \right) , \end{aligned}$$

in which we have assumed that both $G^{(g)}(x)$ and $\widehat{K}^{(g)}(x)$ change slowly with telomere length, *x*. As with (2.6)–(2.7), we aim to solve (2.16) by writing $\widehat{K}^{(g)}(x) = 2^g F(x,\tau )$ where $\tau = Lg$.

The appropriate initial and boundary conditions for *F* are

2.18 $$\begin{aligned}&F(x,0) = H(x-\log (y_0 + y_1Q)) , \qquad F(\log y_0,\tau ) = 0 ,\nonumber \\&\quad \text{ and } \quad F(x,\tau ) \rightarrow 1 \quad \text{ as } \quad x \rightarrow \infty . \end{aligned}$$

The advantage of reformulating the problem in this way is evident in the theory following (2.8) now being applicable.

### Solution of the continuum model

The solution of the pde (2.9) with boundary conditions (2.18) is a moving Gaussian of the form

2.19 $$\begin{aligned} F(x,\tau ) = \frac{1}{2}\left[ 1+\text{ erf }\left( \frac{2x-2\log (y_0)+\tau }{\sqrt{ 2 L \tau \,}} \right) \right] . \end{aligned}$$

This cumulative distribution corresponds to the Gaussian density $f=F_x$ given by

2.20 $$\begin{aligned} f(x,\tau ) = \sqrt{ \frac{2}{\pi L \tau } } \exp \left( - \frac{2}{L\tau } (x-x_0 + \frac{1}{2}\tau )^2 \right) . \end{aligned}$$

Recalling from (2.14) and (2.7) that, to leading order,

2.21 $$\begin{aligned} \widetilde{K}_n^{(g)} \; = \; \widehat{K}^{(g)}(x) \; = \; \frac{\mathrm{d}G^{(g)}}{\mathrm{d}x} \; = \; 2^g f(x,\tau ) \frac{\mathrm{d}x}{\mathrm{d}n}, \end{aligned}$$

we obtain the telomere length distribution in terms of the original variables (*n*, *g*) as

2.22 $$\begin{aligned} \widetilde{K}_n^{(g)} \approx \frac{2^{g+1}\,y_1}{L(y_{0} + y_1n) \sqrt{2\pi g}} \exp \left( -\frac{2}{g} \left[ \frac{1}{L} \log \left( \frac{y_0 + y_1n}{y_0 + y_1Q}\right) + \frac{g}{2} \right] ^2 \right) \, . \end{aligned}$$

**Fig. 2:**
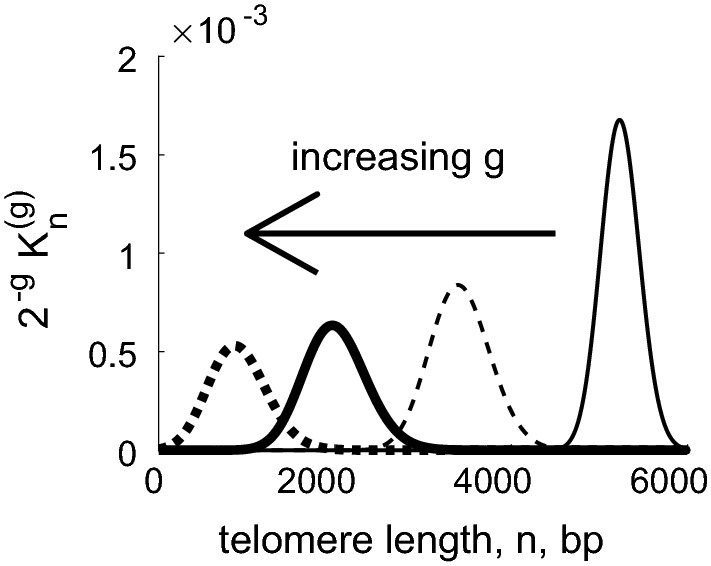
Numerical results obtained from (2.22) showing how, for Case A, the distribution of telomere lengths changes with generation number, *g*. The scaled distribution of telomere lengths $2^{-g} K_n^{(g)}$ broadens and shortens in subsequent generations. Parameter values: $Q=5950$, $y_1=1/60$, $y_0 = 50$. Key: profiles are plotted at generations $g=10$ (narrow solid line), 40 (narrow dashed line), 70 (thick solid line),100 (thick dotted line)

In Fig. 2 we use (2.22) to plot $2^{-g} \widetilde{K}_n^{(g)}$ against *n* for fixed values of *g* in the case $y_1=1/60$, $y_0=50$, $Q=5950$. As expected, the distribution is bell-shaped and widens over time. A more detailed analysis of this figure reveals that the leftward movement slows at later times, and that the distribution is skewed to the right, that is, it is not symmetric when reflected in the mode, and the decay as $n\rightarrow \infty$ is slower than the decay as $n\rightarrow 0$.

Having obtained solutions to the differential Eq. (2.9) which approximate solutions of the discrete model (2.2), we use (2.21) to transform back to the physically meaningful (*n*, *g*)-coordinate systems to interpret and comment on the results. We note that $\widetilde{K}_n^{(g)} / \sum _{n'} \widetilde{K}_{n'}^{(g)}$ is a probability distribution function, with $(y_0+y_1 n)$ having a log-normal distribution, that is, $\log (y_0+y_1n)\sim \mathcal {N}(\widetilde{\mu },\sigma ^2)$ with

2.23 $$\begin{aligned} \widetilde{\mu }(g) = \log (y_0+y_1Q) - \frac{1}{2}g L , \qquad \sigma (g)= \frac{1}{2}L \sqrt{g} . \end{aligned}$$

In terms of the telomere length, *n*, the mode, median, mean and standard deviation of the distribution are

2.24 $$\begin{aligned} n_{\mathrm{mode}}= & {} \frac{ \mathrm{e}^{\widetilde{\mu }-\sigma ^2} - y_0 }{y_1} = \left( Q + \frac{y_0}{y_1} \right) \mathrm{e}^{-Lg/2 - L^2g/4} -\frac{y_0}{y_1}, \end{aligned}$$

2.25 $$\begin{aligned} n_{\mathrm{med}}= & {} \frac{ \mathrm{e}^{\widetilde{\mu }} - y_0}{y_1} = \left( Q + \frac{y_0}{y_1} \right) \mathrm{e}^{-Lg/2} - \frac{y_0}{y_1}, \end{aligned}$$

2.26 $$\begin{aligned} n_{\mathrm{mean}}= & {} \frac{ \mathrm{e}^{\widetilde{\mu }+\sigma ^2/2} }{y_1} = \left( Q + \frac{y_0}{y_1} \right) \mathrm{e}^{-Lg/2 + L^2g/8} -\frac{y_0}{y_1} , \end{aligned}$$

2.27 $$\begin{aligned} n_{\mathrm{sd}}= & {} \frac{\mathrm{e}^{\widetilde{\mu }+\sigma ^2/2} \sqrt{\mathrm{e}^{\sigma ^2}-1} }{y_1} \; = \; \left( Q + \frac{y_0}{y_1} \right) \mathrm{e}^{-Lg/2 + L^2g/8} \sqrt{ \mathrm{e}^{g L^2/4}-1 } \nonumber \\\sim & {} \frac{1}{2}L \sqrt{g} \left( Q + \frac{y_0}{y_1} \right) \mathrm{e}^{-Lg/2} . \end{aligned}$$

Under the assumption that $g=\mathcal {O}(L^{-1})$, we have $n_{\mathrm{med}} = n_{\mathrm{mean}} = n_{\mathrm{mode}}$ to leading order. Including first order correction terms in $L\ll 1$ we have

2.28 $$\begin{aligned} n_{\mathrm{mode}} = n_{\mathrm{med}} - \frac{1}{4} L^2 g ( n_{\mathrm{med}} +y_0/y_1) ,&\;\;\;&n_{\mathrm{mean}} = n_{\mathrm{med}} + \frac{1}{8} L^2 g (n_{\mathrm{med}} + y_0/y_1).\nonumber \\ \end{aligned}$$

The final expression (2.27), which is also obtained using this assumption, shows that the standard deviation increases with generation number for $0<g<1/L$ and decreases for $g>1/L$.

We denote by $K_{\mathrm{tot}}^{(g)}$ the total number of chromosomes at generation *g*, by $\mu _{n}(g)$, the mean telomere length of a chromosome at generation *g*, and by $\phi _{\mathrm{div}}(g)$, the fraction of dividing chromosomes. These quantities are defined by

2.29 $$\begin{aligned} K^{(g)}_{\mathrm{tot}} = \displaystyle \sum _{n=0}^Q \widetilde{K}_n^{(g)} , \quad \mu _{n}(g) = \displaystyle \frac{1}{K_{tot}^{(g)}} \displaystyle \sum _{n=0}^Q n \widetilde{K}_n^{(g)} \, ,\quad \phi _{\mathrm{div}}(g) =\displaystyle \frac{1}{K_{\mathrm{tot}}^{(g)}} \displaystyle \sum _{n=n_c}^Q \widetilde{K}_n^{(g)}.\nonumber \\ \end{aligned}$$

Guided by (2.1), in order for cell division to occur, we require $n>n_c$ (2.1), so that all daughter chromosomes have telomeres above the threshold length. Chromosomes with telomeres of length $0<n<n_c$ exist but cannot replicate; we term these cells ‘senescent’ and note that the fraction of such senescent chromosomes is $\phi _{\mathrm{sen}}(g) = 1 -\phi _{\mathrm{div}}(g)$. These cells contribute to $K^{(g)}_{\mathrm{tot}}$ and $\mu _n(g)$ but not to $\phi _{\mathrm{div}}(g)$.

**Fig. 3:**
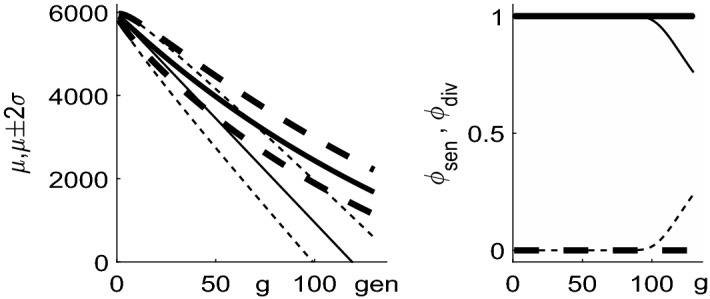
Left: plot of the mean telomere length over 120 generations. The thicker solid line corresponds to the case $Q=5950$, $y_0=50$, $y_1=1/60$ illustrated in Fig. 2, the narrower solid line to the case $y_0=100$, $y_1=0$. In both cases, dotted lines show two standard deviations above and below the mean. Right: the proportion of dividing chromosomes $\phi _{\mathrm{div}}$ (solid lines) decrease over time as the telomeres shorten, and $\phi _{\mathrm{sen}}$ (dashed). For the case $y_1=0$, $y_0=100$, we observe the formation of senescent chromosomes around generation 100

To illustrate features of the distribution of telomere lengths, in Fig. 3 we plot the mean telomere length $\mu _n(g)$ and the fraction of dividing chromosomes $\phi _{\mathrm{div}}(g)$ over many generations. From the left panel of Fig. 3 we note that, in general, the loss of telomere is in general not linear over time.

Before considering Case B for which both telomere loss and the probability of replication are telomere-length dependent, we pause to consider the case for which the number of base pairs lost per replication is constant, that is, $y_1=0$ in (2.22). It is straightforward to show that in place of (2.22) we have

2.30 $$\begin{aligned} \widetilde{K}_n^{(g)} = \frac{2^{g+1}}{y_0 \sqrt{2\pi g}} \exp \left( - \frac{2}{y_0^2 g} \left( Q - n - \frac{1}{2}y_0 g \right) ^2 \right) . \end{aligned}$$

Thus the distribution of telomere lengths is Gaussian, with a mean $\mu _n(t)=(Q-\frac{1}{2}y_0t)$ which decreases linearly with time, and a standard deviation $\sigma _n(t) = \frac{1}{2}y_0 \sqrt{t}$; these results support those presented by Levy et al. (1992), who assumed that the distribution was binomial, and obtained a linear dependence of the mean with time. The proportion of senescent cells is given by

2.31 $$\begin{aligned} \phi _{\mathrm{sen}}(g)=\frac{\sum \limits _{n=0}^{y_0} \widetilde{K}_n^{(g)}}{\sum \limits _{n=0}^Q \widetilde{K}_n^{(g)}} \approx \frac{ \text{ erf }\left( \displaystyle \frac{ 2Q-y_0 g }{y_0\sqrt{2g}} \right) -\text{ erf }\left( \displaystyle \frac{ 2Q-y_0g-2y_0 }{y_0\sqrt{2g}} \right) }{ 1 + \text{ erf }\left( \displaystyle \frac{ 2Q-y_0 g }{ y_0 \sqrt{2g}} \right) } . \end{aligned}$$

In the more general case, where $y_1>0$, the fraction of senescent cells is given by $\phi _{\mathrm{sen}}(g) = \sum _{n=0}^{n_c} K_n^{(g)} / \sum _{n=0}^{Q} K_n^{(g)}$ where $n_c = y_0 / (1-y_1)$, by restricting replication to chromosomes with $n>n_c$ we prevent the formation of physically unrealistic daughter chromosomes with nonpositive telomere lengths.

## Case B: probabilistic chromosomal model of division

### Problem formulation

We now generalise the analysis of the previous section to situations in which the probability of replication is telomere length-dependent. In more detail, we return to the formulation of models of cell division in which generation number *g* is discrete, telomeres are measured in terms of the number of base pairs, *n* and the number of chromosomes at generation *g* with telomere length *n* is denotes by $K_n^{(g)}$.

We suppose that a chromosome whose telomeres have length *n*, divides with probability $P_{\mathrm{div}}(n)= an+b$ where the constants *a* and *b*, are chosen so that $0\le P_{\mathrm{div}}(n) \le 1$. Since $0\le n\le Q$, the relevant restrictions on *a*, *b* are $0\le b \le 1$ and $-b/Q \le a \le (1-b)/Q$. In addition, we assume $P_{\mathrm{div}}(n) =0$ when $n<n_c = y_0/(1-y_1)$.

With $P_{\mathrm{div}}(n)=an+b$ and $y(n)=y_0+y_1n$, the reaction equation for a single chromosome can be written as

3.1 $$\begin{aligned} K_n^{(g)} \rightarrow \left\{ \begin{array}{ll} K_{n}^{(g+1)} +K_{n-(y_0+y_1n)}^{(g+1)} &{} \quad \text{ with } \text{ probability } \ P_{\mathrm{div}}(n) = an + b\,, \\ K_{n}^{(g+1)} &{} \quad \text{ otherwise }. \end{array}\right. \end{aligned}$$

where $0 \le n \le Q$ since the parent chromosome remains in the population regardless of whether replication occurs. Taken together, these replication rules lead to the following equation for the distribution of telomere lengths on generation $(g+1)$

3.2 $$\begin{aligned} \widetilde{K}_{n}^{(g+1)} = \widetilde{K}_n^{(g)} +\left( b + \frac{a(n+y_0)}{1-y_1} \right) \widetilde{K}_{\frac{n+y_{0}}{1-y_{1}}}^{(g)} \, , \end{aligned}$$

which we again solve in $g>0$, for lengths $0 \le n \le Q$, subject to the initial data $K_n^0=\delta _{n,Q}$.

At the start of replication, $n\approx Q$ and the fraction of telomere lost per division event is $y_0/Q + y_1$. By contrast, when *n* is small, replication ends and the fraction of the original telomere length lost per replication is $y_0/Q$. As before, we introduce a small parameter $L \ll 1$ and assume $y_0/Q =\mathcal {O}(L)$ and $y_1 = \mathcal {O}(L)$, so that telomere loss is always small. Under these assumptions, we can perform a Taylor series expansion of the governing Eq. (3.2) in terms of the small parameter, *L*. As in Case A (see (2.4)), we introduce the scalings

3.3 $$\begin{aligned} x= & {} \frac{n}{Q}, \quad (0\le x \le 1) , \quad \tau = L g , \quad L \ll 1 , \quad x, \tau = \mathcal {O}(1) , \end{aligned}$$

3.4 $$\begin{aligned} y_0= & {} QL\widetilde{y}_0, \quad y_1=L\widetilde{y}_1, \quad \widetilde{y}_0, \widetilde{y}_1, a, b = \mathcal {O}(1) . \end{aligned}$$

Following Eq. (2.7), we write

3.5 $$\begin{aligned} \widetilde{K}_n^{(g)} = \xi (\tau ) f(x,\tau ) , \end{aligned}$$

where we assume that the distribution is normalised, *via*$\int _0^1 f(x,\tau ) \, \mathrm{d}x=1$. We define the mean of the distribution $f(x,\tau )$ by

3.6 $$\begin{aligned} \widehat{\mu }(\tau ) = \int _0^1 x f(x,\tau ) \, \mathrm{d}x . \end{aligned}$$

From (3.2), the governing equation is thus

3.7 $$\begin{aligned} \frac{\xi (\tau +L) }{\xi (\tau )} f(x,\tau + L) = f(x,\tau ) + \left( b + \frac{aQ (x+L\widetilde{y}_0)}{1-L\widetilde{y}_1} \right) f\left( \frac{x+L\widetilde{y}_0}{1-L\widetilde{y}_1} , \tau \right) .\nonumber \\ \end{aligned}$$

We expand the delay in the argument of *f* in (3.7), to obtain

3.8 $$\begin{aligned} \frac{\xi (\tau +L) }{\xi (\tau )} f(x,\tau + L)= & {} f(x,\tau ) + \left( b + \frac{aQ (x + L\widetilde{y}_0)}{1-L\widetilde{y}_1} \right) f( x + L \varDelta _1 + L^2 \varDelta _2 , \tau ) , \nonumber \\ \varDelta _1= & {} (\widetilde{y}_0 + \widetilde{y}_1 x) , \qquad \varDelta _2 = \widetilde{y}_1 (\widetilde{y}_0+\widetilde{y}_1 x) . \end{aligned}$$

Performing a Taylor expansion of the last term, $f(x+L\varDelta _1+L^2\varDelta _2)$, to $\mathcal{O}(L^2)$ gives

3.9 $$\begin{aligned} f + L \varDelta _1 f_x + L^2 \varDelta _2 f_x + \frac{1}{2}L^2 \varDelta _1^2 f_{xx} . \end{aligned}$$

We now Taylor expand the small differences in the $\tau$-argument of $f(\cdot ,\cdot )$, also to $\mathcal{O}(L^2)$, to obtain the pde

3.10 $$\begin{aligned} \frac{\xi (\tau +L)}{\xi (\tau ) } \left( f + L f_\tau + \frac{1}{2}L^2 f_{\tau \tau } \right) = \frac{1}{2}L^2 D(x) f_{xx} + L v(x) f_x + {\varGamma (x) f},\qquad \end{aligned}$$

where

3.11 $$\begin{aligned} D(x)= & {} (b+aQx)(\widetilde{y}_0+\widetilde{y}_1 x)^2 , \end{aligned}$$

3.12 $$\begin{aligned} v(x)= & {} (b+aQx)(\widetilde{y}_0+\widetilde{y}_1 x) + L (\widetilde{y}_0+\widetilde{y}_1x) (b\widetilde{y}_1 + aQ\widetilde{y}_0 + 2 a Q \widetilde{y}_1 x) , \qquad \end{aligned}$$

3.13 $$\begin{aligned} {\varGamma (x)}= & {} { (1 + b + aQx) + aQL (\widetilde{y}_0 + \widetilde{y}_1x) + aQL^2\widetilde{y}_1 (\widetilde{y}_0 + \widetilde{y}_1x)}. \end{aligned}$$

Due to the *x*-dependence in the coefficients on the rhs of (3.7), it is not possible to simultaneously remove the terms involving both $f_{\tau \tau }$ and $f_{x\tau }$ by expanding around $(x+\delta L,\tau +\sigma L)$ for some $\delta ,\sigma$. However, our choice of $\delta =0=\sigma$ means that no $f_{x\tau }$ terms are generated.

To find an expression for $\xi (\tau +L)/\xi (\tau )$, we integrate (3.10) with respect to *x*, using (3.6) and $\int f\,\mathrm{d}x=1$. We assume the boundary conditions $f(x)\rightarrow 0$ as $x\rightarrow \pm \infty$, so that $\int v(x) f_x \mathrm{d}x = - \int v'(x) f \mathrm{d}x$ and $\int D(x) f_{xx} \mathrm{d}x = \int D''(x) f \mathrm{d}x$; whereupon we find

3.14 $$\begin{aligned} \xi (\tau + L) = \xi (\tau ) \, \left[ \, 1+b+aQ\widehat{\mu } - L \widetilde{y}_1 (b + a Q \widehat{\mu }) \right] , \end{aligned}$$

This equation can be solved for $\xi (\tau )$, once $\widehat{\mu }(\tau )$ has been determined; this in turn requires some knowledge of $f(x,\tau )$. In practice, we use (3.14) to eliminate $\xi (\tau )$ from (3.10), which yields

3.15 $$\begin{aligned} \theta f_\tau + \frac{1}{2}\theta L f_{\tau \tau } = \frac{1}{2}L D f_{xx} + v f_x + {\varUpsilon } f + L^{-1} a Q (x-\widehat{\mu }) f , \end{aligned}$$

where *D*, *v* are given by (3.11)–(3.12) and

3.16 $$\begin{aligned} \theta= & {} 1 + b + a Q \widehat{\mu } - L\widetilde{y}_1 ( a Q \widehat{\mu } + b ) \equiv \xi (\tau +L) / \xi (\tau ) ,\end{aligned}$$

3.17 $$\begin{aligned} {\varUpsilon }= & {} (b\widetilde{y}_1 + a Q \widetilde{y}_0 + a Q \widetilde{y}_1 x + a Q \widetilde{y}_1\widehat{\mu }) + a Q \widetilde{y}_1 L (\widetilde{y}_0+\widetilde{y}_1 x) . \end{aligned}$$

Although our main focus is on the range $0<x<1$, Eq. (3.15) is well-defined on the whole of $\mathbb {R}$. For simplicity we work with the boundary conditions

3.18 $$\begin{aligned} f(x,\tau ) \rightarrow 0 , \quad \text{ as } \quad x \rightarrow \pm \infty , \end{aligned}$$

since $f(x,0)=0$ for $x>1$ and Eq. (3.15) advects to the left (smaller *x*). Since $f=0$ for $x<0$, we expect $f\rightarrow 0$ as $x\rightarrow -\infty$. Whilst the advection term in Eq. (3.15) will eventually cause $f(x,\tau )$ to become significant in $x<0$, we neglect these since they correspond to the offspring of senescent telomeres and are only formed after the population has become senescent. Hence, we also have $f_x,f_{xx}\rightarrow 0$ as $x\rightarrow \pm \infty$.

In order to specify the initial condition for (3.15), we recall that $K_n^{(0)} = \delta _{Q,n}$, which implies that the cumulative distribution for $K_n^{(0)}$ is $\sum _{n=-\infty }^q K_n^{(0)} = H(q-Q)$. Thus the cumulative distribution corresponding to *f*(*x*, 0), namely $\xi (0) \int ^x_{-\infty } f(x,0)\, \mathrm{d}x$, should be given by $H(x-1)$ and

3.19 $$\begin{aligned} f(x,0) = \delta (x-1) . \end{aligned}$$

Since (3.15) is second order in $\tau$, we formally require two initial conditions. However, this equation is overdamped, and we are mainly concerned with the solutions at later times, which we obtain by solving the first-order problems obtained by truncating the asymptotic expansions in $L \ll 1$. Therefore we specify only (3.19).

In the next subsection, we consider the leading order equation, and derive an exact expression for the mean telomere loss over many generations. By performing a more detailed asymptotic calculation in Sect. 3.3, we derive analytic expressions for the shape of the distribution at early times.

### Analysis of the leading order PDE

To $\mathcal {O}(L)$, Eq. (3.15) reduces to the first-order wave equation

3.20 $$\begin{aligned} (b+aQ\widehat{\mu } +1)f_\tau= & {} (b+a Qx)(\widetilde{y}_0+\widetilde{y}_1 x) f_x + L^{-1} a Q (x - \widehat{\mu })f \nonumber \\&+\, (b\widetilde{y}_1+aQ\widetilde{y}_0+aQ\widetilde{y}_1\widehat{\mu }+aQ\widetilde{y}_1 x) f , \end{aligned}$$

which we solve using the method of characteristics. Since $f(x,0) = \delta (x-1)$,

3.21 $$\begin{aligned} f(x,\tau )=A(\tau )\,\delta \left( A(\tau )\,[x-{\widehat{\mu }}(\tau )]\right) , \end{aligned}$$

and $\widehat{\mu }(\tau )$ is determined by the characteristic that passes through $x=1$ at $\tau =0$:

3.22 $$\begin{aligned} \frac{\mathrm{d}\widehat{\mu }}{\mathrm{d}\tau } = -\frac{(b+aQ\widehat{\mu })(\widetilde{y}_0 + \widetilde{y}_1\widehat{\mu })}{(1+b+aQ\widehat{\mu })} , \qquad \widehat{\mu }(0)=1 . \end{aligned}$$

which can be solved implicitly, for the general case, by

3.23 $$\begin{aligned} \tau (\widehat{\mu }) = \frac{1}{\widetilde{y}_1} \log \left( \frac{\widetilde{y}_0+\widetilde{y}_1}{\widetilde{y}_0+\widetilde{y}_1\widehat{\mu }} \right) + \frac{1}{aQ\widetilde{y}_0-b\widetilde{y}_1} \log \left( \frac{(b+aQ)(\widetilde{y}_0+\widetilde{y}_1\mu )}{(b+aQ\widehat{\mu })(\widetilde{y}_0+\widetilde{y}_1)} \right) . \end{aligned}$$

Expression (3.23) is valid if $\widetilde{y}_1\ne 0$ and $aQ\widetilde{y}_0 \ne b \widetilde{y}_1$. In the special case $\widetilde{y}_1=0$, the solution of (3.22) is

3.24 $$\begin{aligned} \tau (\widehat{\mu })=\frac{1-\widehat{\mu }}{\widetilde{y}_0}+\frac{1}{aQ\widetilde{y}_0} \log \left( \frac{b+aQ}{b+aQ\widehat{\mu }} \right) , \end{aligned}$$

and when $aQ\widetilde{y}_0=b\widetilde{y}_1$, we have

3.25 $$\begin{aligned} \tau (\widehat{\mu }) = \frac{1}{\widetilde{y}_1} \log \left( \frac{b+aQ}{b+aQ\widehat{\mu }} \right) + \frac{a Q (1-\widehat{\mu })}{\widetilde{y}_1 (b+aQ)(b+ aQ\widehat{\mu })} , \end{aligned}$$

neither of which can be recast into explicit expressions for $\widehat{\mu }(\tau )$ with elementary functions.

An approximation of the time to senescence is given by $\tau _{\mathrm{sen}}=\tau (0)$ which, from (3.23), yields

3.26 $$\begin{aligned} \tau _{\mathrm{sen}} = \frac{1}{\widetilde{y}_1} \log \left( 1 + \frac{\widetilde{y}_1}{\widetilde{y}_0} \right) + \frac{1}{aQ\widetilde{y}_0 - b\widetilde{y}_1} \log \left( \frac{ 1 + aQ/b }{ 1 + \widetilde{y}_1/\widetilde{y}_0 } \right) ; \end{aligned}$$

the corresponding expressions for the time of onset of senescence in the special cases $y_1=0$ and $a Q \widetilde{y}_0=b\widetilde{y}_1$ are

3.27 $$\begin{aligned} \tau _{\mathrm{sen}} = \frac{1}{\widetilde{y}_0} +\frac{1}{aQ\widetilde{y}_0} \log \left( 1 + \frac{aQ}{b} \right) , \quad \tau _{\mathrm{sen}} = \frac{1}{\widetilde{y}_1} \log \left( 1 + \frac{aQ}{b} \right) + \frac{a Q}{b\widetilde{y}_1 (b+aQ)},\nonumber \\ \end{aligned}$$

respectively.

The amplitude, $A(\tau )$, in (3.21) solves

3.28 $$\begin{aligned} \frac{\mathrm{d}(\log A)}{\mathrm{d}\tau } =\frac{b\widetilde{y}_1 + aQ\widetilde{y}_0 + 2aQ\widetilde{y}_1\widehat{\mu }}{1+b+aQ\widehat{\mu }} . \end{aligned}$$

Dividing the corresponding sides of (3.28) by (3.22) and integrating with respect to $\widehat{\mu }$ supplies

3.29 $$\begin{aligned} A(\tau ) = A(\tau (\widehat{\mu })) = \frac{(b+ aQ)(\widetilde{y}_0 + \widetilde{y}_1)}{(b+ aQ\widehat{\mu })(\widetilde{y}_0+\widetilde{y}_1\widehat{\mu })} . \end{aligned}$$

Using (3.29) to substitute with $A(\tau )$ in (3.21), we deduce

3.30 $$\begin{aligned} f(x,\tau ) = \frac{(b+ aQ)(\widetilde{y}_0 + \widetilde{y}_1)}{(b+ aQ\widehat{\mu }(\tau ))(\widetilde{y}_0+\widetilde{y}_1\widehat{\mu }(\tau ))} \delta \left( \frac{(b+ aQ)(\widetilde{y}_0 + \widetilde{y}_1) [x - \widehat{\mu }(\tau ) ] }{(b+ aQ\widehat{\mu }(\tau ))(\widetilde{y}_0+\widetilde{y}_1\widehat{\mu }(\tau ))} \right) , \end{aligned}$$

where $\widehat{\mu }(\tau )$ is given implicitly by (3.27) if $y_1=0$ or $a Q \widetilde{y}_0 = b \widetilde{y}_1$ and Eq. (3.23) otherwise.

Returning to (3.14), now with expressions determining the mean $\widehat{\mu }(\tau )$, we evaluate the population size, $\xi (\tau )$, by summing the leading order terms over $0 \le g=\tau '/L \le G-1=\tau /L -1$

3.31 $$\begin{aligned} \log (\xi (\tau ' + L))-\log (\xi (\tau '))=\log ( 1+b+aQ\widehat{\mu }(\tau ') ) , \end{aligned}$$

to obtain

3.32 $$\begin{aligned} \log \xi (\tau )\sim & {} \frac{1}{L} \int _{\tau '=0}^{\tau } \log (1+b+aQ \widehat{\mu }(\tau ')) \, \mathrm{d}\tau ' \nonumber \\= & {} \int _{\mu =\widehat{\mu }(\tau )}^1 \frac{(1+b+ aQ\mu ) \log (1+b+aQ\mu )}{(b+ aQ\mu )(\widetilde{y}_0+\widetilde{y}_1\mu )}\,\mathrm{d}\mu . \end{aligned}$$

In the special case $\widetilde{y}_1=0$, we have

3.33 $$\begin{aligned} \log (\xi (\tau ) )= & {} \frac{1}{aQy_0} \left[ (1 + b + aQ)\log (1 + b + aQ) \right. \nonumber \\&\left. - (1 + b + aQ\widehat{\mu }) \log (1 + b + aQ\widehat{\mu }) \right. \nonumber \\&\left. -\,aQ (1-\widehat{\mu }) + \mathrm{Li}_2(-b-aQ\widehat{\mu }) - \mathrm{Li}_2(-b-aQ) \right] , \end{aligned}$$

where $\mathrm{Li}_2(\cdot )$ is a dilogarithm (Olver et al. 2010; Abramowitz and Stegun 1972) ($\mathrm{Li}_2(z) = -\int _0^z t^{-1} \log (1-t)\mathrm{d}t = \sum _{n=1}^\infty z^n/n^2$).

**Fig. 4:**
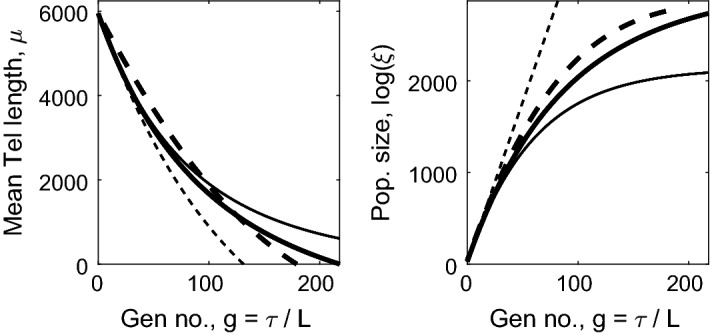
Plots of the mean telomere length $Q\mu (\tau )$ and population size $\xi (\tau )$ as defined by Eqs. (3.23) and (3.32) respectively against generation number $g =\tau /L$ for various choices of $a,b,y_0,y_1$. In both panels: the thick solid line corresponds to $y_0= 50$, $y_1=1/60$, $a=0.8/Q$, $b=0.2$; the thick dashed line corresponds to $y_0=100$, $y_1=0$, $a=0.8/Q$, $b=0.2$; the narrow solid line corresponds to $y_0= 50$, $y_1=1/60$, $a=1/Q$, $b=0$; and narrow dashed lines correspond to $y_0= 50$, $y_1=1/60$, $a=0$, $b=1$

In Fig. 4, we show how, for Case B, the mean $\widehat{\mu }(\tau )$ and the total number of chromosomes $\xi (\tau )$, as defined by Eqs. (3.23) and (3.32), change over time when $L \ll 1$. We note that when $b=1$, $a=0$ we have $P_{\mathrm{div}} =1$ and so recover Case A; when the telomere loss is constant, with $y_0=100$ bp per generation (narrow dashed line) we observe exponential growth of the population, and an approximately linear reduction in average telomere length, confirming the earlier analysis. The thick dashed line, where telomere loss is constant, and $P_{\mathrm{div}} = 0.2 + 0.8 (n/Q)$ also gives approximately linear loss in average telomere length, but now with a rate of population increase which slows at later generations. The two cases for which both telomere loss and replication probability are telomere-length dependent yield population growth curves which slow at later times. Additionally, the rate at which the average telomere length shortens decreases with generation number, and also show average telomere lengths decreasing with time in a nonlinear fashion.

From the first-order pde (3.20), we have found approximations to the average telomere length, $\mu (\tau )$ and the number of chromosomes, $\xi (\tau )$, for the general case where both the probability of replication and amount of telomere lost depend on telomere length (with $L \ll 1$). In order more accurately to describe the distribution in the next subsection we retain second-order derivatives in our pde approximation to the underlying discrete model.

### Early time asymptotic analysis of second-order pde

Our analysis of the (leading order) first-order pde (3.20) supplies expressions for the number of chromosomes, $\xi (\tau )$, and the mean telomere length, $\widehat{\mu }(\tau )$ given by (3.22)–(3.23). Since the distribution is given as a Dirac $\delta$-function, these expressions do not provide information about the variance of the distribution. In order to determine how the distribution evolves over time, we introduce new variables (*z*, *T*) which are chosen to retain the second-order spatial derivative from (3.15). Thus we define

3.34 $$\begin{aligned} x = \widehat{\mu }(\tau ) + L^{2/3} z , \qquad \tau = L^{1/3} T , \end{aligned}$$

so that the typical deviation of telomere lengths from the mean, $x = \widehat{\mu }$ is of magnitude $\mathcal {O}(L^{2/3})$ and $z=\mathcal {O}(1)$. With $T=\mathcal {O}(1)$, this description corresponds to early times, $\tau = \mathcal {O}(L^{1/3})$. We write $f(x,\tau ) = L^{-2/3} \widetilde{f}(z,T)$ so that the initial condition $f(x,0)=\delta (x-1)$ and global constraint $\int f(x,\tau ) \, \mathrm{d}x =1$ imply

3.35 $$\begin{aligned} \widetilde{f}(z,0)= \delta (z) , \qquad \int \widetilde{f}(z,T) \, \mathrm{d}z =1 . \end{aligned}$$

In terms of the new variables, the pde (3.15)–(3.17) becomes

3.36 $$\begin{aligned} \theta \frac{\partial {\widetilde{f}}}{\partial {T}} = \frac{(D-\theta \mu '^2)}{2} \frac{\partial ^2{\widetilde{f}}}{\partial {z}^2} + \left( \frac{ v + \theta \widehat{\mu }'(\tau ) }{L^{1/3}} \right) \frac{\partial {\widetilde{f}}}{\partial {z}} + a Q z \widetilde{f} , \end{aligned}$$

where primes denote derivatives with respect to the argument, and the leading order (in $L\ll 1$) expressions for the parameters are

3.37 $$\begin{aligned} \widehat{\mu }'(\tau )= & {} - \frac{ (b + aQ\widehat{\mu }) (\widetilde{y}_0 + \widetilde{y}_1\widehat{\mu }) }{(1+b+aQ\widehat{\mu })}, \qquad \begin{array}{l} \theta = 1 + b + aQ \widehat{\mu } , \\ D = (b+aQ\widehat{\mu })(\widetilde{y}_0+\widetilde{y}_1\widehat{\mu })^2 , \end{array} \nonumber \\ v= & {} (b + aQ\widehat{\mu })(\widetilde{y}_0 + \widetilde{y}_1\widehat{\mu }) +L^{2/3}(\lambda (T) + z) (aQ\widetilde{y}_0 + b\widetilde{y}_1 + 2aQ\widetilde{y}_1\widehat{\mu }).\nonumber \\ \end{aligned}$$

To satisfy Eq. (3.36) at leading order, we note that $v+\theta \widehat{\mu }'=\mathcal {O}(L^{1/3})$, from (3.22) and the definitions of $\theta ,v$ above. However, there will be some correction term, which, for the simplicity of later calculations, we write as $v + \theta \widehat{\mu }' = L^{1/3} \lambda ''(T)$. The function $\lambda (T)$ will be determined *via* the normalisation condition $\int \widetilde{f} \,\mathrm{d}z=1$. Under these assumptions, Eq. (3.36) reduces to

3.38 $$\begin{aligned} \theta \frac{\partial {\widetilde{f}}}{\partial {T}} = \frac{1}{2}\widetilde{D} \frac{\partial ^2{\widetilde{f}}}{\partial {z}^2} + \lambda ''(T) \frac{\partial {\widetilde{f}}}{\partial {z}} + a Q z \widetilde{f} , \end{aligned}$$

where $\widetilde{D} = D - \theta \mu '^2$. Since $T=\mathcal {O}(1)$ corresponds to an initial short timescale, in which $\tau =\mathcal {O}(L^{1/3})$, the mean telomere length is given by $\widehat{\mu }(\tau )=1$. In this case, the coefficients in (3.38) take the constant values $\theta = 1+b+aQ$, $D=(b+aQ) (\widetilde{y}_0+\widetilde{y}_1)^2$ and $\widetilde{D} = (b+aQ)(\widetilde{y}_0 +\widetilde{y}_1)^2 / (1+b+aQ)$.

Taking the Fourier transform of (3.38) with $\widehat{f}(k,T) =\int \mathrm{e}^{ikz} \widetilde{f}(z,T)\,\mathrm{d}z$, we obtain

3.39 $$\begin{aligned} \theta \frac{\partial {\widehat{f}}}{\partial {T}} + a Q i \frac{\partial {\widehat{f}}}{\partial {k}} =\left[ - \frac{1}{2}\widetilde{D} k^2 - i k \lambda ''(T) \right] \widehat{f} , \end{aligned}$$

where the initial data and global constraint (3.35) imply $\widehat{f}(k,0)=1$, and $\widehat{f}(0,T)=1$. The general solution of (3.39) is

3.40 $$\begin{aligned} \widehat{f}(k,T)= & {} \widehat{C}(aQT + i\theta k) \nonumber \\&\times \exp \left( \frac{\widetilde{D} a^2 Q^2 T^3}{6 \theta ^3} +\frac{ i \widetilde{D} a Q T^2 k}{2\theta ^2} -\frac{ \widetilde{D} T k^2}{2 \theta } -\frac{ i k \lambda '(T)}{\theta } -\frac{ a Q \lambda (T)}{\theta ^2}\right) ,\nonumber \\ \end{aligned}$$

where $\widehat{C}(\cdot )$ is an arbitrary function and, without loss of generality, we assume $\lambda (0)=0=\lambda '(0)$.

The initial condition and global constraint (3.35) imply $\widehat{C}(q)\equiv 1$ and $\lambda (T) = \widetilde{D} a Q T^3 / 6 \theta$ so that the solution of the Fourier Transform Eq. (3.39) is given by $\widehat{f}(k,T) = \exp ( - \widetilde{D} T k^2 / 2\theta )$. Inverting the transform *via*$\widetilde{f}(z,T) =(2\pi )^{-1} \int \mathrm{e}^{-ikz} \widehat{f}(k,T) \mathrm{d}k$ leads to

3.41 $$\begin{aligned} \widetilde{f}(z,t) = \frac{\sqrt{\theta }}{\sqrt{2\pi \widetilde{D} T}} \, \exp \left( - \frac{\theta \, z^2 }{2 \widetilde{D} T} \right) . \end{aligned}$$

This distribution is a Gaussian pulse which converges to a Dirac $\delta$-function as $T\rightarrow 0^+$, and which spreads with the standard $z \sim \sqrt{T}$ scaling as $T\rightarrow \infty$.

Recalling (3.37), we obtain

**Fig. 5:**
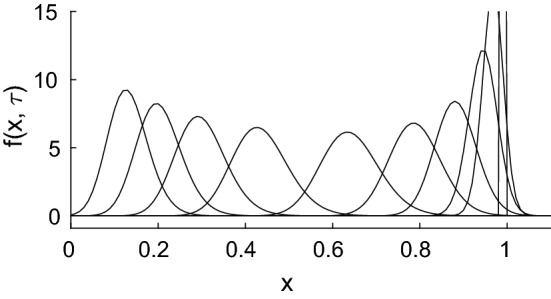
Illustration of the numerical solution of (3.15) in the case $L=0.01$, $Q=5950$, $a=0.8/Q$, $b=0.2$, $y_0=50$, $y_1=1/60$, plotted at $\tau = 0$ (cut off), 0.005, 0.01, 0.02, 0.05, 0.1, 0.2, 0.4, 0.6, 0.8, 1.0

3.42 $$\begin{aligned} f(x,\tau ) = \frac{(1+b+aQ)}{(\widetilde{y}_0+\widetilde{y}_1)\sqrt{2\pi L \tau (b+aQ) }} \, \exp \left( - \frac{(1+b+aQ)^2 \, (x-\widehat{\mu }(\tau ))^2 }{2 L \tau (b+aQ)(\widetilde{y}_0+\widetilde{y}_1)^2 } \right) ,\nonumber \\ \end{aligned}$$

which is valid for $\tau = \mathcal {O}(L^{1/3})$ and $x-\widehat{\mu }(\tau )=\mathcal {O}(L^{2/3})$, where $\widehat{\mu }(\tau ) \sim 1 - \tau (b+aQ)(y_0+y_1)/(1+b+aQ)$. Over the longer timescale ($\tau = \mathcal {O}(1)$), the distribution becomes skewed as shown in the numerical solution presented in Fig. 5, which is due to the effect of the *x*-dependence of the coefficients (3.11)–(3.17), in (3.15).

### Summary

In Sects. 2 and 3 we derived two models for the evolution of telomere lengths over time in a population of replicating chromosomes. We used asymptotic methods to determine the shape of the distribution in various cases. For Case A, all chromosomes replicate, with probability one on each generation, provided their telomeres are sufficiently long. and the distribution has a Gaussian or a log-normal shape. For Case B, the probability of chromosome replication depends on telomere length. We obtain an expression for the evolution of the mean for all times, and show that at early times, the distribution is Gaussian; however, the scaling for the dependent variables (3.34) is nonstandard.

The analyses for Cases A and B describe a population of chromosomes with each replicating independently. In practice, this description is overly simplistic: cells containing multiple ($N=46$) chromosomes replicate and the checkpoints on replication occur at the cell level. Hence in the next section we incorporate this level of complexity and consider a population of cells, each of which contains *N* chromosomes.

## Case A: deterministic cell-level model of normal ageing

In this section, we upscale the model from Sect. 2, which describes populations of chromosomes replicating independently, to investigate cell populations, in which each cell contains *N*-chromosomes. Our model describes the distribution of telomere lengths of the chromosomes within each cell. In this section we consider the fully deterministic Case A, and in Sect. 5 we consider the more general probabilistic model, Case B, from Sect. 3.

We will assume that a cell can only replicate if all of its $N=46$ chromosomes have telomeres which exceed the threshold length; if the length of *any* telomere falls below the threshold, the cell becomes senescent and it will not replicate further, although it will remain a viable member of the cell population. During cell division, each chromosome replicates, producing two daughter chromosomes, which are allocated to the two daughter cells randomly and independently of the other replicating chromosomes. In contrast to the models presented in Sects. 2 and 3, it is now highly likely that the total telomere length of each daughter cell will be less than that of their parent. We consider the same rules for chromosome replication as in (1.1); we use *m*, to denote the *total* telomere length in the cell and *Y*(*m*), to denote the *total* amount of telomere lost during each replication event.

### Development of discrete cell-scale model

As in (2.2), when a cell divides, each chromosome produces one daughter chromosome with the same telomere length as the parent and a second with shorter telomere length. When specifying replication rules, we number chromosomes in a cell using *r*, with $1\le r \le N$. Following (1.1), we write

4.1 $$\begin{aligned} K^{(r,g)}_{n_r} \rightarrow K^{(r,g+1)}_{n_r} +K^{(r,g+1)}_{n_r-y(n_r)} , \end{aligned}$$

where $n_r$ denotes the telomere length of the $r^{\mathrm{th}}$ chromosome ($1\le r \le N$) in a given cell, *g* represents the generation number, and *y*, the amount of telomere lost from one daughter chromosome upon replication. By analogy with (1.2) we define $y(n_r)=y_0+Ny_1n_r$, where $y_0,y_1$ are constants; this form is chosen to simplify later calculations. Combining *N* chromosomes into one cell, and describing the state of each cell solely by its *total* telomere length, *m*, and generation number, *g*, we define

4.2 $$\begin{aligned} C_m^{(g)} = \bigcup _{r=1}^N K_{n_r}^{(r,g)} , \quad \text{ where } \quad m = \sum _{r=1}^N n_r , \quad (0\le m \le NQ). \end{aligned}$$

with the aim of summing (4.1) over *N* chromosomes, to form a replication rule for cell $C_m^{(g)}$.

We now simplify the telomere loss rule. A model that includes information on the telomere length of each chromosome in a cell would not be analytically tractable. Since we propose to only retain information on the total telomere length of the cell, we assume telomere loss *Y*(*m*) depends only on the total telomere length in the cell, *m*. Thus, for each chromosome (*r*), we write

4.3 $$\begin{aligned} Y(m) = y_0 + m y_1, \end{aligned}$$

which replaces the term $y(n_r)$ in (4.1). To confirm this expression is consistent with the individual loss term $y(n_r) = y_0 + N y_1 n_r$ , we calculate the maximum total loss of telomere, which is given by *NY*(*m*), and

4.4 $$\begin{aligned} NY(m) = \sum _r y(n_r) = \sum _r [ y_0 + N y_1 n_r ] = N y_0 + N y_1 \sum _r n_r = N (y_0 + y_1 m) . \end{aligned}$$

If we assume that shortening of the *r*th chromosome can be inherited by either daughter cell, then there are $2^N$ different ways of allocating the longer and shorter daughter chromosomes to the two daughter cells. If we assume that each arrangement is equally likely, then we have

4.5 $$\begin{aligned} C_m^{(g)} \rightarrow C_{m - jY(m)}^{(g+1)} +C_{m - (N - j)Y(m)}^{(g+1)} , \ (0 \le j \le N) \ \ \text{ with } \text{ probability } \;\; \displaystyle \frac{1}{2^N} \left( \begin{array}{c} N\\ j \end{array}\right) .\nonumber \\ \end{aligned}$$

by combining (4.1) with (4.2). The number of shortened chromosomes a daughter cell inherits determines the amount by which its telomere length is reduced, this can vary between zero and *NY*(*m*) bps. Cells with telomere length *m* can be the offspring of cells with telomere length $(m+jy_0) / (1-jy_1)$ for any $0\le j \le N$. Summing (4.5) over all possibilities, weighted according to the corresponding probabilities, gives a discrete evolution equation for $C_m^{(g)}$, the number of cells at generation *g* with total telomere length *m*,

4.6 $$\begin{aligned} C_{m}^{(g+1)} = \frac{1}{2^N} \sum _{j=0}^N \left( \begin{array}{c} N\\ j \end{array}\right) \left[ C_{\frac{m+jy_0}{1-jy_1}}^{(g)} +C_{\frac{m+(N-j)y_0}{1-(N-j)y_1}}^{(g)} \right] \, = \frac{2}{2^N} \sum _{j=0}^N \left( \begin{array}{c} N\\ j \end{array}\right) C_{\frac{m+jy_0}{1-jy_1}}^{(g)}.\qquad \end{aligned}$$

The simplification is due to the summand being invariant under the transformation $j\mapsto N-j$. Typically, we solve this model subject to the initial conditions $C_m^{(0)} = \delta _{m,NQ}$, which corresponds to a single cell with total telomere length $m=NQ$ at generation zero.

### Deterministic continuum cell-level model

We start by reformulating (4.6) in terms of the cumulative distribution function $G_m^{(g)}$ which satisfies $C_m^{(g)} = G_m^{(g)} - G_{m-1}^{(g)}$, which is equivalent to $G_m^{(g)}=\sum _{q=0}^m C_q^{(g)}$. Hence

4.7 $$\begin{aligned} G_{m}^{(g+1)} = \frac{2}{2^N} \sum _{j=0}^N \left( \begin{array}{c} N\\ j \end{array}\right) G_{\frac{m+jy_0}{1-jy_1}}^{(g)} . \end{aligned}$$

If we permit telomeres of negative lengths to form (i.e. $m<0$), then the number of telomeres doubles every generation and the boundary conditions for the cumulative distribution function are

4.8 $$\begin{aligned} G_m^{(g)}\rightarrow & {} 0 \qquad \text{ as } \;\;\; m \rightarrow -\infty , \nonumber \\ G_m^{(g)}\rightarrow & {} 2^g \qquad \text{ as } \;\;\; m \rightarrow \infty , \end{aligned}$$

since, at the start of the simulations we have one cell with total telomere given by $m=m_0 = NQ$. To determine the shape of the distribution, we rescale to remove the exponential growth by writing $G_m^{(g)} = 2^g F(x,\tau )$, where $x=x(m)$ and $\tau = L g$ are new independent variables.

The dimensionless small parameter, *L*, is defined by the small initial relative rate of loss of telomere, that is

4.9 $$\begin{aligned} \frac{ N (y_0 + y_1 NQ)}{NQ} =: L \ll 1 . \end{aligned}$$

We introduce rescaled parameters

4.10 $$\begin{aligned} y_0=LQ\widetilde{y}_0,\qquad y_1=\frac{L\widetilde{y}_1}{N}, \qquad \widetilde{y}_0, \widetilde{y}_1,N = \mathcal {O}(1) , \end{aligned}$$

Following (2.14), we replace the discrete variable *m* (with $0 \le m \le NQ$) by the continuous variable *x*, where

4.11 $$\begin{aligned} x=\log (QN\widetilde{y}_0+m\widetilde{y}_1), \quad \log (QN\widetilde{y}_0) \le x\le x_{\mathrm{max}}:=\log (NQ(\widetilde{y}_0+\widetilde{y}_1)).\qquad \end{aligned}$$

It has already been noted that cells with telomere length $\widetilde{m} = (m+jy_0)/(1-jy_1)$ give rise to daughters of length *m*; correspondingly, daughter cells with parameter *x* arise from parents with parameter $\widetilde{x}$ given by

4.12 $$\begin{aligned} \widetilde{x} = x - \log \left( 1 - \frac{j \widetilde{y}_1L}{N} \right) \sim x + \frac{j \widetilde{y}_1L}{N} + \frac{j^2 y_1^2L^2}{N^2} . \end{aligned}$$

where we have used $\log (1-h)\sim -h-\frac{1}{2}h^2$ for $h\ll 1$. Combining the asymptotic scalings (4.10) with the change of variable (4.11), the expansion (4.12), $\tau =Lg$ and $G_m^{(g)}=2^g F(x,\tau )$, Eq. (4.7) implies

4.13 $$\begin{aligned}&F\left( x-\frac{\widetilde{y}_1 L}{4},\tau +\frac{L}{2}\right) \nonumber \\&\quad =\sum _{j=0}^N 2^{-N} \left( \begin{array}{c} N\\ j \end{array}\right) F\left( x - \frac{\widetilde{y}_1 L}{4} + \frac{j\widetilde{y}_1 L}{N} +\frac{j^2 \widetilde{y}_1^2 L^2}{N^2} , \tau - \frac{L}{2} \right) . \end{aligned}$$

The discrete differences in this model can, with good accuracy, be replaced by derivatives; using Taylor’s series, based on $L\ll 1$ and $j\widetilde{y}_1L/N\ll 1$. Here we have introduced the shifts $x\mapsto x-\frac{1}{4} \widetilde{y}_1 L$ ($\delta = \mathcal {O}(1)$) and $\tau \mapsto \tau -\frac{1}{2}L$ so that when we perform the Taylor series expansion in $L \ll 1$, the coefficients of $F_{\tau \tau }$ or $F_{x\tau }$ vanish. Thus we obtain

4.14 $$\begin{aligned} F_\tau = v F_x + D F_{xx} , \quad v = \frac{1}{2}\widetilde{y}_1 +\frac{1}{4} L \widetilde{y}_1^2 \left( 1 + \frac{1}{N}\right) , \quad D = L \widetilde{y}_1^2/N. \end{aligned}$$

We solve (4.14) subject to the initial conditions $F(x,0) = H(x-x_{\mathrm{max}})$, where $H(\cdot )$ is the Heaviside function, and the boundary conditions $F(x,\tau )\rightarrow 0$ as $x\rightarrow -\infty$ and $F(x,\tau ) \rightarrow 1$ as $x\rightarrow +\infty$ which together imply

4.15 $$\begin{aligned} F(x,\tau ) = \frac{1}{2}\left[ 1+ \text{ erf }\left( \frac{x+v\tau -x_{\mathrm{max}}}{2\sqrt{D\tau }} \right) \right] . \end{aligned}$$

Equation (4.15) corresponds to the cumulative distribution function for a Gaussian distribution which has density

4.16 $$\begin{aligned} f(x,\tau ) = \frac{\partial F}{\partial x} =\frac{1}{2\sqrt{\pi D \tau }} \exp \left( -\frac{(x+v\tau -x_{\mathrm{max}})^2}{4D\tau }\right) . \end{aligned}$$

Rewriting (4.16) in terms of telomere length, *m*, and generation number, *g*, we deduce that the distribution of telomere lengths is given by a log-normal distribution. In more detail, we have $C_m^{(g)} = 2^g (\partial F/\partial x)(\mathrm{d}x/\mathrm{d}m)$, so that

4.17 $$\begin{aligned} C_m^{(g)} = \frac{2^{g+1} }{ (y_0+y_1m) \sqrt{2\pi Ng}} \exp \left( - \frac{2}{ N y_1^2 g } \left[ \log \left( \frac{y_0+y_1m}{y_0+NQy_1} \right) + \frac{1}{2}N y_1 g \right] ^2 \right) .\nonumber \\ \end{aligned}$$

In the special case $y_1=0$, we define $x=m/NQ$ so that $0\le x \le 1$, and in place of (4.12), we have $\widetilde{x} =x + j y_0/NQ = x + j \widetilde{y}_0 L/N$. When $y_1=0$, Eqs. (4.14) and (4.17) are replaced by

4.18 $$\begin{aligned} \frac{\partial {F}}{\partial {\tau }} = \frac{\widetilde{y}_0}{2} \frac{\partial {F}}{\partial {x}} +\frac{L\widetilde{y}_0^2}{8N} \left( 1 + \frac{1}{N} \right) \frac{\partial ^2{F}}{\partial {x}^2} , \end{aligned}$$

and

4.19 $$\begin{aligned} C_m^{(g)} = \frac{2^{g+1}}{y_0 \sqrt{2\pi N g} } \exp \left( - \frac{2}{N g y_0^2}\left[ m - NQ + \frac{1}{2}y_0 N g \right] ^2 \right) , \end{aligned}$$

which gives a standard Gaussian distribution, with a mean that reduces linearly over time, and a standard deviation which increases with $\sqrt{g}$.

The solutions (4.19) and (4.17) are similar to the chromosome-level results (2.22), which are plotted in Fig. 3. Before illustrating the behaviour of the cell-level model, we explore the distribution of telomere lengths within each cell so as to determine when senescence occurs.

### Determination of time to senescence

A cell will not divide if the telomere length of any of its chromosomes falls below the critical length, here taken to be zero. To determine when a cell reaches senescence, we must determine the length of its *shortest* telomere. We now develop a method for approximating the length of the shortest telomere in a cell so that our model can predict the time of the onset of senescence.

In each cell there is a subpopulation of *N* telomeres, whose lengths at generation *g* we define by $\{S^{(g)}_j\}_{j=1}^N$. Since daughter telomeres are randomly allocated to daughter cells, the length of each telomere $S_j^{(g)}$ is effectively given by a random number *R* / *N* where *R* is drawn from the distribution $C_m^{(g)} / \sum _m C_m^{(g)}$. If we randomly pick a sample of *N* such variables, label them $\{r_1,\ldots ,r_N\}$ and then order them so that $x_i=r_j$ for some permutation such that $x_1< x_2< x_3 \ldots < x_N$, then $x_1=\min _j \{r_j\}$. The minimum of the sample $x_1$ is distributed according to the first order statistic.

**Fig. 6:**
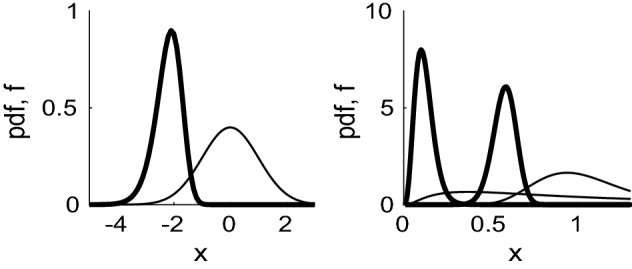
Left: probability distribution function (pdf) for the Gaussian, N(0,1), (narrow line, given by (4.21), defined for all *x*), and that of the lowest order statistic of $N=46$ chromosomes (thicker line), given by (4.20). Right: similar plots for the log-normal distribution where $\log X \sim N(\mu ,\sigma )$ with $\mu =0$ ($f=\exp (-(\log x - \mu )^2 / 2\sigma ^2)/x\sigma \sqrt{2\pi }$, which is only defined for $x>0$). The pdf (thin line) for the case $\sigma =1$ has a maximum near $x=0.4$ and the pdf of the first order statistic (4.20) has a maximum near $x=0.1$; the case $\sigma =0.25$ has a pdf with maximum just below $x=1$ and the first order statistic has a maximum around $x=0.5$

Given the probability density function *f*(*r*) and the cumulative distribution function (cdf) *F*(*r*) for the variables $r_j$, the probability density function $f_1$ and cumulative distribution function $F_1$ of the first-order statistic $x_1=\min _j \{ r_j \}$ (Hogg and Craig 1970) are

4.20 $$\begin{aligned} f_{1}(x_{1}) = N ( 1-F(x_{1}) )^{N-1} f(x_{1}) \, , \quad F_{1}(x_1) = 1 - (1 - F(x_1) )^N . \end{aligned}$$

To illustrate, we consider a Gaussian distribution where the pdf and cdf of a $N(\mu ,\sigma )$ are given by

4.21 $$\begin{aligned} f(r) = \frac{1}{\sigma \sqrt{2\pi }} \exp \left( - \frac{(r-\mu )^2}{2\sigma ^2} \right) \, , \quad F(r) = \frac{1}{2} \left( 1 + \text{ erf }\left( \frac{r-\mu }{\sigma \sqrt{2}} \right) \right) \, . \end{aligned}$$

In the left panel of Fig. 6, the narrow line represents the pdf *f*(*r*) for the case of zero mean and unit standard deviation, *N*(0, 1). The thicker line represents the distribution of the first order statistic, $f_1(x_1)$ given by (4.21), that is, $x_1=\min _j \{r_j\}$ and $\{r_j\}_{j=1}^{N}$ are $N=46$ sample random variables sampled from the distribution *f*(*r*) (4.20). The distribution of the first order statistic $f_1(x_1)$ is noticeably shifted to the left when compared to *f*(*r*). It also skewed and has a smaller variance.

The mean of the first order statistic can be calculated as $\mathbb {E} [ f_{(1)}(x_{1})] = -2.216$. Whilst the standard deviation of the normalised Gaussian is unity, that of the first order statistic with $N=46$ is 0.469—significantly smaller than that for the population as a whole. The mode of the pdf of the first order statistic, $f_1(x_1)$, is given by the maximum of $f_1(x_1)$, which occurs when $f'_1(x_1)=0$, that is

4.22 $$\begin{aligned} \frac{x_1}{2}\left( 1-\text{ erf }\left( \frac{x_1}{\sqrt{2}} \right) \right) = - \frac{(N-1)}{\sqrt{2\pi }} \mathrm{e}^{-x_{1}^2/2} \,. \end{aligned}$$

Solving Eq. (4.22) numerically in the case $N=46$ yields $x_1=-2.084$. The median of the distribution $f_{(1)}(x_1)$ is given by $F_{(1)}(x_1)=\frac{1}{2}$, which implies $\text{ erfc }(x_1/\sqrt{2}) = 2^{1-1/N}$; solving this equation numerically, gives the value $x_1=-2.171$ when $N=46$. Thus, the median and mode are similar, but not identical, to the mean value. To summarise, for a Gaussian distribution of telomere lengths, the length of the shortest of $N=46$ telomeres is just over two standard deviations below the mean.

The right-hand panel of Fig. 6 illustrates two cases of a log-normal distribution, where $\log X \sim N(0,1)$ and $\log X \sim N(0,\frac{1}{4})$. Given $\log X \sim N(\mu ,\sigma )$, we have $\mathbb {E}[X] = \mathrm{e}^{\mu +\sigma ^2/2}$ and $\mathbb {V}[X] =\mathbb {E}[X^2]-\mathbb {E}[X]^2 = \mathrm{e}^{2\mu +\sigma ^2} (\mathrm{e}^{\sigma ^2}-1)$. The smaller value of $\sigma$ gives rise to a distribution with a smaller mean, that is more skewed, and correspondingly, a smaller first order statistic.

**Fig. 7:**
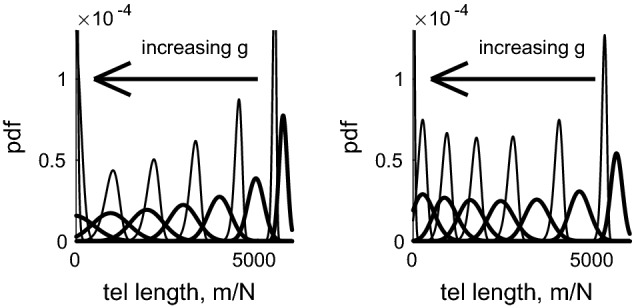
Left, thick lines show the pdf of telomere lengths $C_m^{(g)} / \sum _m C_m^{(g)}$ from (4.19), plotted against average telomere length *m* / *N*, at generations $g=5$, 20, 40, 60, 80,100, 120, for the case $y_0=100$, $y_1=0$. Narrow lines illustrate the pdf of the first order statistic (4.21) of $N=46$ chromosomes. Right, similar but for $y_0=50$, $y_1=1/60$ with $C_m^{(g)}$ given by (4.17). To allow comparison with Figs. 2 and 3, the horizontal axis has been scaled to show the average telomere length, that is, the total telomere length divided by the number of chromosomes ($N=46$)

**Fig. 8:**
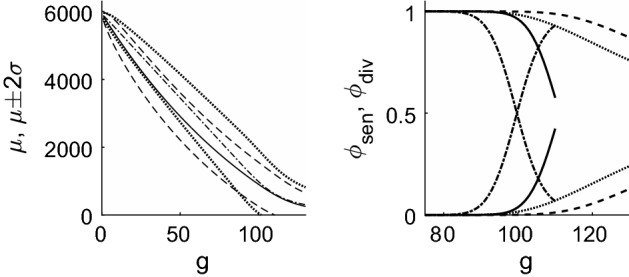
Left: plots of mean telomere length against generation number for the case $y_0=100$, $y_1=0$ in dash-dotted line, with the dotted lines showing mean ± 2 s.d. The case $y_0=50$, $y_1=1/60$ is shown with a solid line, with mean $\pm 2$ s.d. shown by dashed lines. To allow comparison with Figs. 2 and 3, the vertical axis has been scaled to show the *average* telomere length, that is, the total telomere length divided by the number of chromosomes ($N=46$). Right: the proportion of dividing ($\phi _{\mathrm{div}}$) and senescent ($\phi _{\mathrm{sen}} = 1 -\phi _{\mathrm{div}}$) chromosomes/cells plotted against generation number, *g*. The dashed line corresponds to the chromosome level model with $y_0=50$, $y_1=1/60$; the dotted line corresponds to the chromosome level model with $y_0=100$, $y_1=0$; the solid line corresponds to the cell level model with $y_0=50$, $y_1=1/60$; the dash-dotted line corresponds to the cell level model with $y_0=100$, $y_1=0$

We now return to the full solution for telomere length distributions (4.17) and (4.19). Figures 7 and 8 show that when $y_1=0$, the telomere length decreases over time at a uniform rate; when $y_1>0$ the shortening rate reduces in the later generations. We note also that when $y_1=0$, the distribution spreads considerably, whilst when $y_1>0$, the increase in variance almost ceases at later times. This is particularly noticeable in the behaviour of the first order statistic shown by the narrower lines in Fig. 7.

The fraction of dividing and senescent cells over time is shown in Fig. 8, where the curves which decrease from unity as *g* increases correspond to $\phi _{\mathrm{div}}(g)$, namely the dividing fraction and those that increase from zero show the senescent proportion, $\phi _{\mathrm{sen}}(g)$. The chromosome-level definition of senescence is given by Eq. (2.31) and the text immediately following it; for the cell-level description, we define the pdf of total telomere length by $f(m,g) = C_{m}^{(g)} / \sum _{m=0}^{NQ} C_{m}^{(g)}$, with the cumulative distribution function being given by $F(m,g) = \sum _{j=0}^m f(j,g)$. We next compute the pdf of the first order statistic, as $f_1(m,g) = N f(m,g) (1-F(m,g))^{N-1}$ using (4.20), then the fraction of senescent cells is given by $\phi _{\mathrm{sen}}(g) = \sum _{m=0}^{m_c} f_1(m,g)$ where $m_c =Ny_0/(1-y_1)$. We denote $\phi _{\mathrm{div}}(g) = 1-\phi _{\mathrm{sen}}(g)$. From Fig. 8 we note that the cell-level definition of senescence gives a much more abrupt transition from dividing population to senescence, and that this occurs at a slightly earlier time than predicted by the chromosome-level models.

## Case B: model with length-dependent division probability

### Development of continuum cell-level model

In this section we upscale the probabilistic model from Sect. 3 to a cell-level description. We suppose that the amount of telomere lost per replication and the probability of replication depend on telomere length. The probability replication is $P_{\mathrm{div}} = am+b$ where *a*, *b* are constants chosen to ensure that $0 \le P_{\mathrm{div}} \le 1$. The amount of telomere lost per chromosome is $y_0 + m y_1$, so the discrete cell replication rule can be written as

5.1 $$\begin{aligned} C_{m}^{(g)}\longrightarrow & {} (1 - am - b)C_{m}^{(g+1)} \nonumber \\&+\sum _{j=0}^N 2^{-N} \left( {\begin{array}{c}N\\ j\end{array}}\right) (am + b) \left[ C_{m-j(y_0+my_1)}^{(g+1)} + C_{m-(N-j)(y_0+my_1)}^{(g+1)} \right] ,\qquad \end{aligned}$$

Since, after summing over *j*, the two terms in square brackets generate identical contributions, for simplicity we take double the first term.

In order to determine which parental cells in generation *g* with total telomere lengths $m'$ yield a daughter cell in generation $(g + 1)$with total telomere length *m*, we replacing *m* in (5.1) by $m+Y_j$; Eq. (5.1) can then be simplified to

5.2 $$\begin{aligned} C_{m+Y_j}^{(g)}\longrightarrow & {} (1 - am - b - aY_j) C_{m+Y_j}^{(g+1)} \nonumber \\&+\sum _{j=0}^N 2^{1-N} \left( {\begin{array}{c}N\\ j\end{array}}\right) (am + b + aY_j) C_{m+Y_j - jy_0 - jy_1(m+Y_j)}^{(g+1)} \, . \end{aligned}$$

We now choose those $Y_j$ which result in the subscript $m+Y_j -jy_0 - jy_1(m+Y_j) = m$, which implies

5.3 $$\begin{aligned} Y_j= & {} \frac{j(y_0 + y_1m)}{1-jy_1} \nonumber \\= & {} \frac{ L Q j ( \widetilde{y}_0 + \widetilde{y}_1 m / NQ) }{ 1 - \widetilde{y}_1 L j / N } \; \sim \; L Q j \left( \widetilde{y}_0 + \frac{\widetilde{y}_1 m}{NQ} \right) \left( 1 + \frac{L j\widetilde{y}_1}{N} \right) . \end{aligned}$$

Here, following (3.4) and (4.10), we make use of the scalings

5.4 $$\begin{aligned} L \ll 1 , \qquad y_0 = L Q \widetilde{y}_0 , \qquad y_1 = L\widetilde{y}_1 / N , \qquad \widetilde{y}_0,\widetilde{y}_1,N = \mathcal {O}(1) . \end{aligned}$$

From (5.2) with $Y_j$ given by (5.3) the evolution equation for the number of cells, $C_m^{(g)}$, of telomere length *m* at generation *g* as

5.5 $$\begin{aligned} C_m^{(g+1)}= & {} (1 - am - b) C_m^{(g)} + \sum _{j=0}^N 2^{1-N} \left( \begin{array}{c} N\\ j \end{array}\right) \widetilde{P}_{\mathrm{div}} C_{m + LQj(\widetilde{y}_0+\widetilde{y}_1m/NQ)(1+Lj\widetilde{y}_1/N)}^{(g)} . \nonumber \\ \widetilde{P}_{\mathrm{div}}= & {} b + a m + a LQ j \left( \widetilde{y}_0 +\frac{\widetilde{y}_1 m}{NQ} \right) \left( 1 + \frac{Lj\widetilde{y}_1}{N} \right) . \end{aligned}$$

We now introduce new variables, *x* and $\tau$ defined by

5.6 $$\begin{aligned} m = N Q x,\qquad \tau = L g,\qquad C_m^{(g)}=\xi (\tau ) f(x,\tau ) , \end{aligned}$$

so that $0 \le x \le 1$ and $\tau =\mathcal {O}(1)$ is the timescale over which cells transit from their initial state to senescence. The assumed form for the state variable $C_m^{(g)}$ separates the behaviour into two components: $\xi (\tau )$ which accounts for the total size of the population and is expected to exhibit rapid growth; and $f(x,\tau )$, which describes the shape of the telomere length distribution. By inserting the ansatz (5.6) into (5.5), we obtain the discrete difference equation

5.7 $$\begin{aligned} \theta f(x,\tau + L)= & {} (1-b-aNQx) f(x,\tau ) +\sum _{j=0}^N 2^{1-N} \left( \begin{array}{c} N\\ j \end{array}\right) \nonumber \\&\times \left[ b + aNQx + \frac{a L j (\widetilde{y}_0 + \widetilde{y}_1x)}{N} \left( 1 + \frac{j L \widetilde{y}_1}{N}\right) \right] \nonumber \\&\times f \left( x + \frac{Lj(\widetilde{y}_0 + \widetilde{y}_1 x)}{N} \left( 1 + \frac{L\widetilde{y}_1j}{N}\right) , \tau \right) , \end{aligned}$$

where $$\theta = \xi (\tau +L) / \xi (\tau )$$.

To separate the determination of $\xi (\tau )$ and $f(x,\tau )$, we impose a normalisation condition on *f* and define the mean of the distribution by

5.8 $$\begin{aligned} \int f(x,\tau ) \, \mathrm{d}x = 1 , \qquad \widehat{\mu }(\tau ) := \int x f(x,\tau ) \, \mathrm{d}x . \end{aligned}$$

Then we have that

5.9 $$\begin{aligned}&\int f_x \mathrm{d}x = 0 , \qquad \int x f_x \mathrm{d}x = -1 , \qquad \int x^2 f_x \mathrm{d}x = - 2\widehat{\mu } , \nonumber \\&\int f_{xx} \mathrm{d}x = 0 , \quad \int x f_{xx} \mathrm{d}x = 0 , \quad \int x^2 f_{xx} \mathrm{d}x = 2 , \quad \int x^3 f_{xx} \mathrm{d}x = 6 \widehat{\mu }.\qquad \end{aligned}$$

Integrating (5.7) with respect to *x* and using (5.8)–(5.9), we obtain

5.10 $$\begin{aligned} \theta (\tau ) = \frac{\xi (\tau +L)}{\xi (\tau )} = (1+b+aNQ\widehat{\mu }) - L\widetilde{y}_1 (b+aNQ\widehat{\mu }) . \end{aligned}$$

We now expand (5.7) using Taylor series with $L\ll 1$, and substituting in (5.10), we obtain

5.11 $$\begin{aligned}&\left[ 1 + (b + aNQ\widehat{\mu }) (1 - L\widetilde{y}_1) \right] \left( f_\tau + \frac{1}{2}L f_{\tau \tau } \right) \nonumber \\&\quad = D L f_{xx} + v f_x +\frac{ a NQ( x-\widehat{\mu }) f }{L} + {\widetilde{\varUpsilon }} f , \end{aligned}$$

where

5.12 $$\begin{aligned} {\widetilde{\varUpsilon }}= & {} b \widetilde{y}_1 + a NQ( \widetilde{y}_0 +\widetilde{y}_1 x + \widetilde{y}_1\widehat{\mu } ) + \frac{1}{2}L a \widetilde{y}_1 Q (N + 1) (\widetilde{y}_0 + \widetilde{y}_1 x) , \end{aligned}$$

5.13 $$\begin{aligned} v= & {} (b + aNQx)(\widetilde{y}_0 + \widetilde{y}_1 x)\nonumber \\&+\frac{1}{2}L (\widetilde{y}_0 + \widetilde{y}_1 x) \left( 1 + \frac{1}{N}\right) [ b \widetilde{y}_1 + a Q N \widetilde{y}_0 +2 a Q N \widetilde{y}_1 x ] , \end{aligned}$$

5.14 $$\begin{aligned} D= & {} \frac{1}{4} (b+aNQx) (\widetilde{y}_0+\widetilde{y}_1 x)^2 \left( 1 + \frac{1}{N}\right) . \end{aligned}$$

As with the model considered in Sect. 3, this pde cannot be solved explicitly, so we first consider the first order approximating pde and then the early time asymptotics.

### Analysis of probabilistic cell model

Ignoring the $\mathcal {O}(L)$ terms in (5.11), we obtain a first order pde

5.15 $$\begin{aligned} (1 + b + aNQ\widehat{\mu } ) f_\tau - v f_x =\frac{ a NQ( x-\widehat{\mu }) f }{L} + {\widetilde{\varUpsilon }} f , \end{aligned}$$

which can be solved by the method of characteristics to obtain the following approximate equation for the mean

5.16 $$\begin{aligned} \frac{\mathrm{d}\widehat{\mu }}{\mathrm{d}\tau }= - \frac{(b+aNQ\widehat{\mu }) (\widetilde{y}_0+\widetilde{y}_1\widehat{\mu })}{1 + b + aNQ \widehat{\mu }} . \end{aligned}$$

Imposing $\widehat{\mu }(0)=1$, this ode has the implicit solution

5.17 $$\begin{aligned} \tau (\widehat{\mu })= & {} \frac{1}{ b \widetilde{y}_1 - a NQ \widetilde{y}_0}\nonumber \\&\times \left[ \widetilde{y}_1 \log \left( \frac{b + aNQ\widehat{\mu }}{b+aNQ} \right) + (\widetilde{y}_1 + b\widetilde{y}_1 - aNQ\widetilde{y}_0) \log \left( \frac{\widetilde{y}_0+\widetilde{y}_1}{\widetilde{y}_0 + \widetilde{y}_1 \widehat{\mu }} \right) \right] .\qquad \end{aligned}$$

If $$y_1=0$$, then

5.18 $$\begin{aligned} \tau (\widehat{\mu })=\frac{1-\widehat{\mu }}{\widetilde{y}_0} + \frac{1}{\widetilde{y}_0 aNQ} \log \left( \frac{b+aNQ}{b+aNQ\widehat{\mu }} \right) , \end{aligned}$$

and if $aNQ\widetilde{y}_0=b\widetilde{y}_1$, then

5.19 $$\begin{aligned} \tau (\widehat{\mu }) = \frac{1}{\widetilde{y}_1} \log \left( \frac{b+aNQ\widehat{\mu }}{b+aNQ} \right) - \frac{aNQ(1-\widehat{\mu })}{(b+aNQ)(b+aNQ\widehat{\mu })} . \end{aligned}$$

Following Sect. 3.3, we obtain an approximate expression for the shape of the distribution at early times by introducing the rescalings

5.20 $$\begin{aligned} \tau = L^{1/3} T, \qquad x = \mu (\tau ) + L^{2/3} z , \qquad f(x,\tau ) = L^{-1/3} \widetilde{f}(z,T) , \end{aligned}$$

with $\widehat{\mu }=1$. Then (5.11) supplies

5.21 $$\begin{aligned} (1+b+aNQ) \widetilde{f}_T = \widetilde{D} \widetilde{f}_{zz} + \frac{\widetilde{f}_z}{L^{1/3}} \left[ v +(1+b+aNQ) \frac{\mathrm{d}\mu }{\mathrm{d}\tau } \right] + aNQ z \widetilde{f} , \end{aligned}$$

where the effective diffusivity $\widetilde{D}$ is given by

5.22 $$\begin{aligned} \widetilde{D} = D - \frac{1}{2}(1+b+aNQ) \mu '^2 . \end{aligned}$$

We aim to solve (5.21) subject to the conditions

5.23 $$\begin{aligned} \int \widetilde{f} \,\mathrm{d}z = 1, \quad \int z \widetilde{f} \, \mathrm{d}z = 0 , \quad \widetilde{f}(z,0) = \delta (z) , \quad \widetilde{f}\rightarrow 0 \quad \text{ as } \quad z \rightarrow \pm \infty .\qquad \end{aligned}$$

As with Eqs. (3.36), (3.38), we note that the leading order terms in (5.21), namely those in the square brackets cancel, leaving

5.24 $$\begin{aligned} (1+b+aNQ) \widetilde{f}_T = \widetilde{D} \widetilde{f}_{zz} + \lambda ''(T) \widetilde{f}_z + aNQ z \widetilde{f} , \end{aligned}$$

where $\lambda ''(T)$ represents the (as yet unknown) first correction term from the term in square brackets. If one includes a term of the form $\lambda _2(T)z\widetilde{f}_z$, then the constraints (5.23) imply that $\lambda _2=0$, so we omit such a term from the following analysis. Taking Fourier transforms ($\widehat{f}(k,T)=\int \mathrm{e}^{ikx}\widetilde{f}(z,T) \,\mathrm{d}z$), we obtain

5.25 $$\begin{aligned}&(1 + b + aNQ)\widehat{f}_T + iaNQ\widehat{f}_k = - (\widetilde{D} k^2 + i k \lambda ''(T) )\widehat{f} , \nonumber \\&\quad \widehat{f}(k,0) = 1 , \quad \widehat{f} (0,T) = 1 , \end{aligned}$$

which has the solution

5.26 $$\begin{aligned} \widehat{f}(k,T) =\exp \left( -\frac{\widetilde{D} T k^2}{1 + b + aNQ} \right) , \end{aligned}$$

where, without loss of generality, we impose $\lambda (0)=0=\lambda '(0)$ giving $\lambda (T) = a N Q \widetilde{D} T^3 / 3 (1+b+aNQ)$, hence

5.27 $$\begin{aligned} \widetilde{f}(z,T) = \sqrt{\frac{\pi (1 + b + aNQ)}{\widetilde{D} T }} \exp \left( - \frac{z^2(1 + b + aNQ)}{4\widetilde{D} T} \right) , \end{aligned}$$

and

5.28 $$\begin{aligned} f(x,\tau )=\frac{\pi (1 + b + aNQ)}{L^{1/6} \sqrt{\widetilde{D} \tau }} \exp \left( - \frac{(x-\mu )^2 (1 + b + aNQ)}{4 L \widetilde{D} \tau }\right) . \end{aligned}$$

We note how the generalisation to multiple chromosomes per cell changes the rates at which the distribution of telomere lengths shortens and spreads out. To compare these effects, we introduce two quantities: a loss rate, which gives the rate at which the mean of the telomere length distribution reduces over time, and a ‘diffusivity’, corresponding to the rate at which the distribution widens in terms of the telomere length variable; note that this is not a literal spreading out in physical space. Firstly, we note that the advection terms are very similar for multiple and single chromosomes ($N=1$): defining the loss rate in (3.15)–(3.12) by $v/\theta$ and similarly in (5.11) and (5.13)–(5.14), we have

5.29 $$\begin{aligned} v_{\mathrm{chromo}} = \frac{ (b+aQx)(\widetilde{y}_0+\widetilde{y}_1x) }{(1+b+aQ\mu )} , \qquad \text{ and } \quad v_{\mathrm{cell}} = \frac{(b+aQNx) (\widetilde{y}_0+\widetilde{y}_1x)}{(1+b+aQN\mu )} , \end{aligned}$$

which can be made identical under the mapping $a\mapsto aN$. Next, we compare the rate at which the distribution spreads in the cell model with that in the chromosome model. We find this by applying the transformation (5.20) and then examine the coefficient of $f_{zz}$ in he In the cell model, the diffusivity is given by

5.30 $$\begin{aligned} \mathcal {D}_{\mathrm{cell}}=\frac{D-\frac{1}{2}(1+b+aQN)\mu '^2}{1+b+aQN}, \end{aligned}$$

with $D,\mu '$ given by (5.14), (5.16) respectively, differs from the corresponding quantity in the chromosome model, where $\mathcal {D}_{\mathrm{chromo}} =(D-\theta \mu '^2)/2\theta$ with $D,\theta ,\mu '$ given by (3.16), (3.11), (3.22). Evaluating these expressions at $x=\widehat{\mu }$, we have

5.31 $$\begin{aligned} \mathcal {D}_{\mathrm{chromo}}= & {} \frac{(b + aQ\widehat{\mu }) (\widetilde{y}_0 + \widetilde{y}_1\widehat{\mu })^2}{2(1+b+aQ\widehat{\mu })^2} ,\nonumber \\ \mathcal {D}_{\mathrm{cell}}= & {} \frac{(b + aNQ\widehat{\mu }) (\widetilde{y}_0 + \widetilde{y}_1\widehat{\mu })^2}{2(1+b+aQN\widehat{\mu })^2} \left[ \frac{ N+1}{2N} - \frac{(N-1)(b+aNQ\widehat{\mu }) }{2N} \right] .\qquad \qquad \end{aligned}$$

In the case $N=1$, the expression for $\mathcal {D}_{\mathrm{cell}}$ reduces to $\mathcal {D}_{\mathrm{chromo}}$. Even after applying the mapping $a\mapsto aN$, we see that for $N>1$, $\mathcal {D}_{\mathrm{cell}} < \mathcal {D}_{\mathrm{chromo}}$, due to the mixing of longer and shorter offspring chromosomes into the daughter cell caused by (5.1), in which the dominant terms occur for $j \approx N/2$. The effect of having multiple chromosomes in each cell, and randomly allocating daughter chromosomes to each daughter cell is to *reduce* the relative spread of telomere lengths, which leads to a more abrupt transition from replication to senescence.

**Fig. 9:**
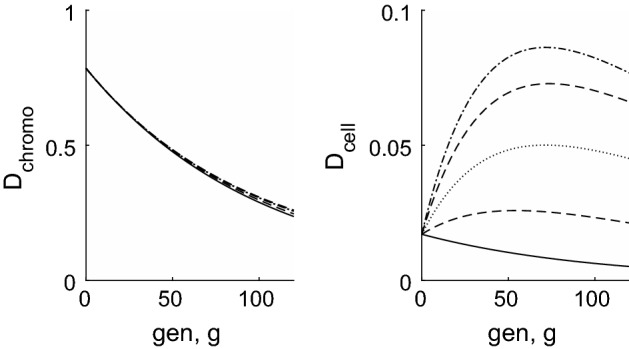
Left: plots of $\mathcal {D}_{\mathrm{chromo}}$ (5.31) against generation number, *g*, for the case of $y_0=50$, $y_1=1/60$; the cases $(a,b)=(0,1)$, (0.2 / *Q*, 0.8), (0.5 / *Q*, 0.5), (0.8 / *Q*, 0.2), (1 / *Q*, 0.0) are illustrated respectively by solid line, dashed line, dotted line, dashed line, dash-dotted line. Right: similar for, $\mathcal {D}_{\mathrm{cell}}$

**Fig. 10:**
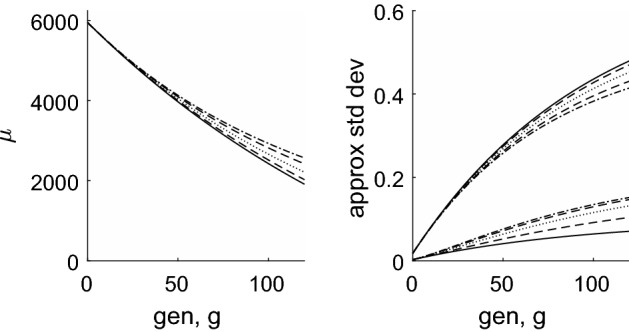
Left: graph of mean telomere length against generation number; right, approximate standard deviation of telomere lengths plotted against generation number; for the same parameter values as used in Fig. 9

In Fig. 9, we use (5.31) to plot both $\mathcal {D}_{\mathrm{chromo}}$ and $\mathcal {D}_{\mathrm{cell}}$ against generation number *g*. The chromosomal case shows little dependence on parameters, and reduces from about 0.8 to approximately 0.2. The diffusivity in the cellular case is much smaller ($\mathcal {D}_{\mathrm{cell}}<0.1$) and, for most of the parameter values illustrated in Fig. 9, $\mathcal {D}_{\mathrm{cell}}$ increases over the early generations and decreases at later times. The initial increase is due to the term in square brackets in (5.31), which is approximately $\frac{1}{2}(1-P_{\mathrm{div}})$. Given $\mathcal {D}_{\mathrm{chromo}}$ and $\mathcal {D}_{\mathrm{cell}}$, we can create a proxy for the standard deviation of the distribution, $\sigma$, by noting that $(\mathrm{d}/\mathrm{d}\tau )(\sigma ^2) = \mathcal {D}$, hence $\sigma =\surd \int _0^\tau \mathcal {D} \, \mathrm{d}\tau$. This expression yields the curves plotted in Fig. 10. On the left, against generation number we plot the average telomere length of a chromosome in base pairs, $Q\mu$. On the right, we plot the approximate standard deviation $\int \sqrt{\mathcal {D}} \, \mathrm{d}\tau$, to illustrate the significant difference between the cellular and chromosomal models, and smaller differences caused by changing the parameters *a*, *b*.

## Discussion

In this paper, we have considered the dynamics of telomere loss in a population of independent chromosomes and a population of cells, each containing a population of chromosomes. Our aim has been to understand the kinetics of telomere loss and onset of senescence, which are hallmarks of aging. During each replication event some telomere is lost from one daughter chromosome, and senescence occurs when the telomere length becomes too short for replication to occur. Since telomere length plays an important role in cell division, we have considered a variety of models, which focus on both the amount of telomere lost, and the rate (probability) of cell division depending on telomere length.

We have developed fully discrete models, and approximated them by deriving continuum level pde descriptions. This procedure relies on only small fraction of total telomere length being lost in each generation, so that an asymptotic reduction yields governing equations both at the level of individual chromosomes, and at the cell-level, where each cell contains a subpopulation of *N* chromosomes. As an example, we consider human cells, where $N=46$. We have analysed these models to determine the evolution of the telomere length distribution over many replication cycles (generations). In all cases we observe a shortening of telomeres, until senescence occurs. In cell models senescence occurs when the *shortest* telomere can no longer undergo replication.

In summary, we have outlined simpler models, which describe a population of individual chromosomes, and more complicated, cell-level, models in which each cell is assumed to contain a subpopulation of $N=46$ chromosomes, and upon replication each chromosome divides, giving one offspring to each daughter cell. In both cases, we have considered *deterministic* models as Case A, where replication occurs each generation, provided senescence has not yet been triggered; and, Case B, a *stochastic* model in which replication occurs with a probability that may depend on telomere length. In each of these four subcases, there is then the possibility that telomere loss is fixed, or dependent on telomere length. We summarise our results in increasing complexity:

- In Case A, when telomere loss is constant, the mean telomere length decreases linearly with generation number, until senescence is reached. This occurs in both the chromosome-level and the cell-level models. In both cases, the distribution of telomere lengths is Gaussian (normal) and with a standard deviation which increases with the square root of the generation number.
- In Case A and where the telomere loss is dependent on telomere length, the mean telomere length still reduces, albeit in an exponential rather than a linear fashion being given by (2.24)–(2.26). The distribution has the form of a shifted log-normal (2.22). The two forms of Case A are compared in Fig. 3 for the chromosome level model.
- Various parameter values of the stochastic replication model, Case B, are illustrated in Fig. 4. In all cases the reduction in telomere length is not constant, and not given by a simple exponential, rather by the more complicated implicit formula (3.23). In this case considering the simpler case of a size-independent telomere loss rate ($y_1=0$) does not simplify the governing Eq. (3.20). The population size grows subexponentially in this case. The distribution for Case B appears almost Gaussian, however, Fig. 5 shows that it is slightly skewed. In this case the pde describing the shape of the length distribution (3.20) of telomeres does not admit explicit analytical solution. However, an approximation enables us to determine the mean telomere length. Through further use of asymptotic techniques, we show that at very early times, the distribution is Gaussian, however, at later times, it becomes skewed.
- The main distinction between the results of the chromosome-level model and the cell-level model is that the latter gives a sharper transition between the whole population being in a replicative state and senescence, as illustrated in Fig. 8.

In all cases, we observe that the distribution of telomere lengths evolves in a manner consistent with corresponding Monte Carlo simulations presented previously (Qi et al. 2014), where fits to the experimental data of Zhang et al. (2000) are illustrated. By varying the telomere loss or replication probability parameters, mean telomere length reduction can be tuned to fit a variety of experimental data.

Chromosome-level models similar to our Cases A and B have been considered by other authors. When Levy et al. (1992) studied a special case of Case A, for which telomere loss is constant (independent of telomere length). By assuming that the number of deletions occurring increased with the number of generations following a binomial distribution, they found the mean number of deletions to increase linearly over time, and obtained a sharp transition from replicative to the senescent state. The model of Buijs et al. (2004) is broadly similar to the more general form of Case A that we use. They fitted the model to an experimental distribution of telomere lengths, verifying the length-dependent loss. Our analysis extends this work by showing that the shape of the distribution is log-normal. Portugal et al. (2008) considered the probability of a cell’s replication being linearly dependent on telomere length—as in our Case B—but with constant amount of telomere lost per replication. Our work extends this to the case of length-dependent loss, and we have also considered the fraction of senescent chromosomes.

As far as we are aware, no existing models consider the distribution of telomere lengths within individual cells. We have extended all these models to consider a population of cells, each of which contains a subpopulation of $N=46$ chromosomes. In these cell-level models, the longer and shorter daughter chromosomes from replication are randomly allocated to the daughter cells. Whilst some cells inherit predominantly longer chromosomes, and a few shorter chromosomes, the majority of cells inherit approximately equal numbers of longer and shorter daughter chromosomes. The overall effect of this is to reduce the variance of total telomere length in the cell-level model (compared to the individual chromosome description, which can be thought of as a cell-level model with $N=1$). Analysis of cell-level continuum models gives some results similar to the individual chromosome model. The mean of the distribution is in good agreement with the Monte Carlo simulations of cell-level models presented previously (Qi et al. 2014). However, the transition between replicative and senescent states is sharper in the cell-level model than in the chromosome model, as noted by Qi et al. (2014). This feature is caused by the length of the shortest telomere in a cell having a smaller variance than the telomere length of overall population, as shown in Fig. 6. Since a cell becomes senescent when its *shortest* telomere cannot replicate, the total telomere length of senescent cells will increase with the number of chromosomes per cell. In the analysis of the cell-level model we have used the theory of order statistics to determine the expected time at which the shortest telomere reaches the threshold of zero length and hence determined the time at which the onset of senescence occurs. We expect the cell-level results presented here to be the same as if we simulated a population of individual chromosomes and then randomly allocated the telomeres into groups of $N=46$. This is because we have assumed a random allocation of long and short daughter telomeres into each cell, as given by (4.7). However, in the case of stem cells, for example, the allocation of long and short telomeres is not random, and so (4.7) would not be valid, and should be replaced with an alternative, which would result in different outcomes.

In future work, we plan to generalise these results to include the effect of telomerase - an enzyme which lengthens telomeres and so allows cells to continue replicating (Qi 2011; Qi et al. 2019); the possibility of varying activity levels of telomerase was discussed by Epel et al. (2004). It may also be possible to generalise the models proposed here to include other mechanisms which influence telomere length, such as the Alternative Lengthening of Telomeres (ALT), telomerase activity, telomere recombination, and Werner’s syndrome—a disease in which accelerated aging is caused by the corrupted replication of chromosomes. This is caused by the failure to resolve stalled forks in the DNA replication process. Muraki et al. (2012) discuss the repair mechanism for double-stranded breaks, and other problems that can occur at the microscopic level, for example, the role of deficiencies of the Shelterin proteins, and the interplay between stochastic telomere loss mechanisms and chromosome instability. Oxidative stress is known to accelerate aging at the cellular level, and many other environmental factors have been shown to correlate with telomere length. Starkweather et al. (2013) discuss the effects of various psychosocial factors on telomere length, including chronic psychological stress, sleep quality, socioeconomic status, educational attainment and genetic components. The mathematical modelling of how these macroscopic factors influence telomere loss at the microscale remains an open problem.
